# Dual-Bioorthogonal Catalysis by a Palladium Peptide Complex

**DOI:** 10.1021/acs.jmedchem.2c01689

**Published:** 2023-02-23

**Authors:** Ana M. Pérez-López, Adam Belsom, Linus Fiedler, Xiaoyi Xin, Juri Rappsilber

**Affiliations:** † Chair of Bioanalytics, Technische Universität Berlin, 10623 Berlin, Germany; ‡ Si-M/“Der Simulierte Mensch”, a Science Framework of Technische Universität Berlin and Charité—Universitätsmedizin Berlin, 10623 Berlin, Germany; § Wellcome Centre for Cell Biology, University of Edinburgh, Edinburgh EH9 3BF, U.K.

## Abstract

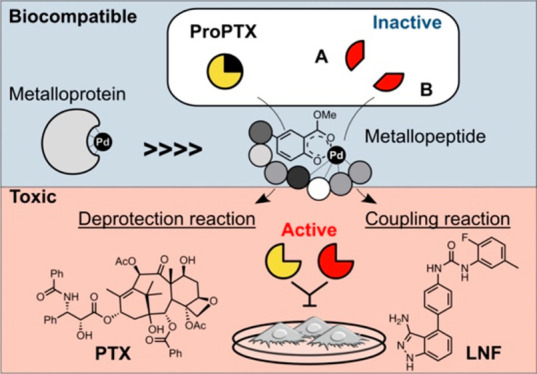

Artificial metalloenzymes (ArMs) enrich bioorthogonal chemistry with new-to-nature reactions while limiting metal deactivation and toxicity. This enables biomedical applications such as activating therapeutics *in situ*. However, while combination therapies are becoming widespread anticancer treatments, dual catalysis by ArMs has not yet been shown. We present a heptapeptidic ArM with a novel peptide ligand carrying a methyl salicylate palladium complex. We observed that the peptide scaffold reduces metal toxicity while protecting the metal from deactivation by cellular components. Importantly, the peptide also improves catalysis, suggesting involvement in the catalytic reaction mechanism. Our work shows how a palladium-peptide homogeneous catalyst can simultaneously mediate two types of chemistry to synthesize anticancer drugs in human cells. Methyl salicylate palladium LLEYLKR peptide (**2-Pd**) succeeded to simultaneously produce paclitaxel by depropargylation, and linifanib by Suzuki–Miyaura cross-coupling in cell culture, thereby achieving combination therapy on non-small-cell lung cancer (NSCLC) A549 cells.

## Introduction

Nature has evolved to use relatively few metals to conduct biological reactions in living systems (Fe, Zn, Ni, Cu, Mg, and Mn). However, the range of possible chemical reactions could be greatly increased if abiotic transition metals could be used.^1^ Since Bertozzi demonstrated that artificial chemical reactions can safely take place in living systems, bioorthogonal chemistry has been used to activate sensors and drugs,^2−5^ repair tissues,^6^ label biomolecules,^7−11^ and modulate biological functions.^12−14^ The use of transition-metal catalysts (TMC) to mediate bioorthogonal local activation of drugs has emerged as a potential type of anticancer treatment, increasing tolerability, and therefore effectiveness, of chemotherapeutics.^15−17^

Bioorthogonal catalysis still has key challenges remaining, including metal toxicity and catalytic yield.^18^ Furthermore, most bioorthogonal TMC are heterogeneous, relying on polymeric supports (polystyrene resins,^4,15,16^ micelles,^19^ hydrogels,^20^ nanoreactors,^21,22^ nanozymes,^23,24^ and metal–organic frameworks (MOFs)^25,26^), which can limit their application for treating solid tumors. Ways to overcome these limitations have employed biological delivery systems, including exosomes^17^ and macrophages,^27^ to encapsulate metals and avoid toxicity. Additionally, heterogeneous catalysts tend to have lower catalytic yield than homogeneous catalysts.^28^

Organometallic complexes of Ru, Au, Pd, or Pt have been explored as homogeneous TMC to mediate bioorthogonal chemistry in cells, including alkyl deprotections,^29^ hydroarylations,^30^ cross-coupling ligations,^31^ isomerisations,^32^ and metathesis.^33^ However, the standard ligands for complexation (e.g., phosphines^34^ or *N*-heterocyclic carbenes^35^ for palladium) are small-sized molecules and the metal can be leached easily by cellular proteins.^36^ Such protein–metal interactions not only are a major cause of toxicity^37^ but also lead to rapid deactivation of the metals catalytic properties.^34^ To solve this problem, polymer-based homogeneous TMC have been applied *in vitro*;^38−43^ however their questionable biocompatibility means that low catalyst concentrations have to be used.

Proteins have been used as biocompatible supports for metals, mimicking the active site of metalloenzymes and forming artificial metalloenzymes (ArMs).^44^ However, there are few examples of ArMs as bioorthogonal, homogeneous, TMC in living systems, due to their prohibitive macromolecular size.^45−47^ Reducing the protein structure to small peptides could overcome large structure limitations, which include delivery issues, *in situ* metal–protein assembly, and immunogenicity.^47,48^

Metallopeptides are an exciting and highly appealing new type of bioorthogonal TMC, achieving homogeneous catalysis with minimal metal toxicity.^49,50^ Here, we have synthesized a novel, bioorthogonal, homogeneous palladium peptide catalyst, consisting of a methyl salicylate tagged hydrophilic peptide (LLEYLKR) complexed to palladium. We then explored its catalytic properties in the context of cultured human non-small-cell lung cancer (NSCLC) A549 cells to demonstrate that simultaneous dual catalysis is possible.

## Results and Discussion

### Pd-Peptide Design and Synthesis

**Figure 1:**
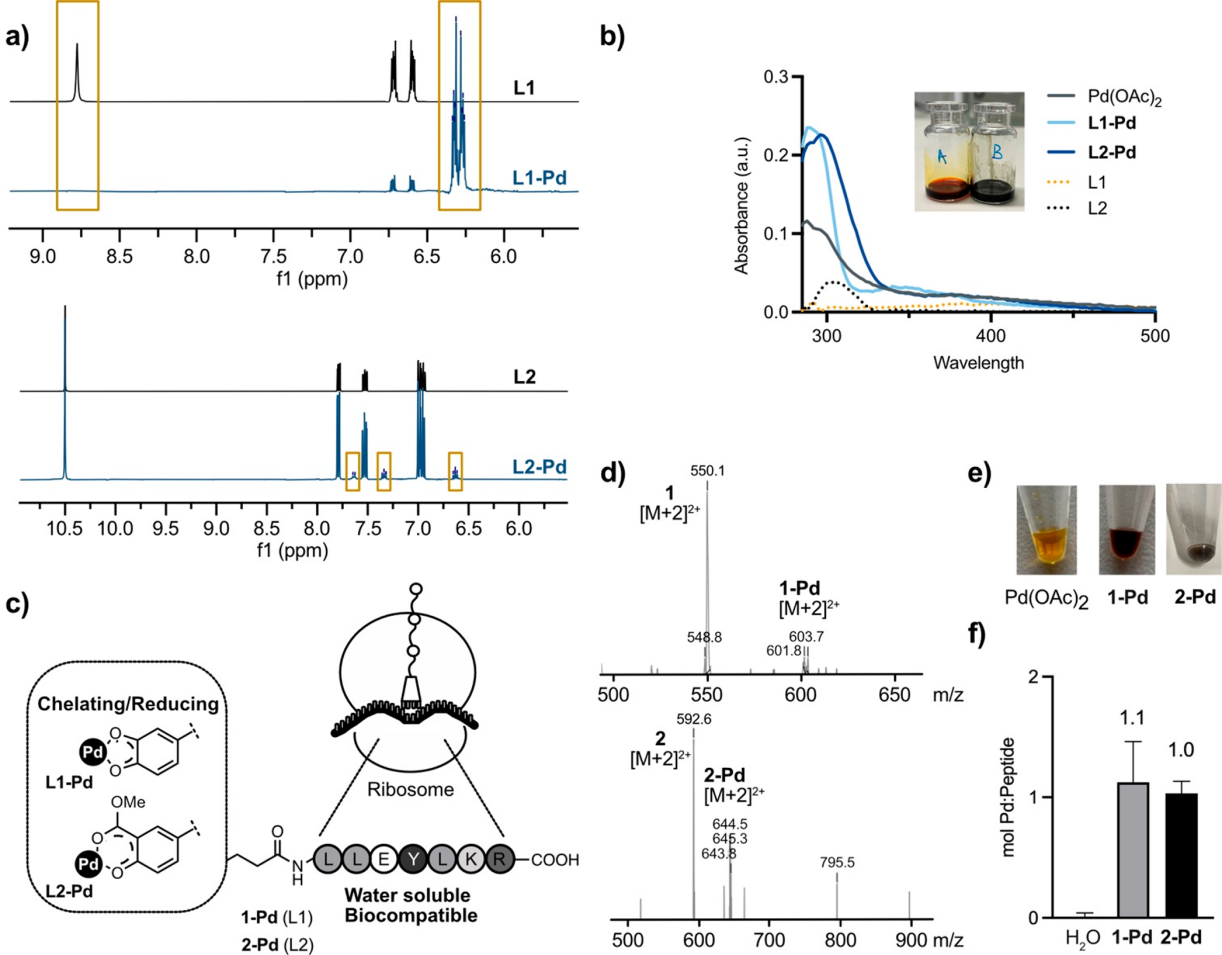
(a) ^1^H NMR of pure 1,2-dihydroxybenzene (**L1**) and methyl salicylate (**L2**) (upper spectra of each set), and complex of **L1** or **L2** with Pd(OAc)_2_ in a 1:1 molar ratio incubated at 37 °C for 1 h in 0.5 mL of DMSO-*d*_6_ (50 mM) (lower spectra of each set). NMR was tested at rt. (b) UV–visible spectra of 1,2-dihydroxybenzene (**L1**), methyl salicylate (**L2**), Pd(OAc)_2_, and the Pd complexes (**L1-Pd** and **L2-Pd**) in a molar ratio 1:1 ligand:Pd(OAc)_2_ (all samples at 100 μM) after incubation at 37 °C for 2 h in PBS (1 mL), as well as darkened color of **L1-Pd** (right vial) upon redox compared to Pd(OAc)_2_ (left vial). (c) Representation of the bioorthogonal homogeneous catalyst. (d) Mass spectrum of metallopeptides **1-Pd** and **2-Pd** matching the [M + 2]^2+^. (e) Color change of **1-Pd** and **2-Pd** after complexation. (f) ICP-OES analysis of the resulting metallopeptides **1-Pd** and **2-Pd** (100 μM). The molar ratio of Pd to peptide after purification is plotted.

Salicylic acid and catechol are well-known chelating agents for palladium.^51−55^ For example, catechol not only coordinates Pd(II) but also reduces and stabilizes Pd(0) species by forming *o*-quinone complexes.^56^ We tested the metal chelating/reducing properties of catechol (**L1**) and methyl salicylate (**L2**), as these could be coupled in a peptide scaffold as metal binding sites. After incubation with Pd(OAc)_2_ in deuterated DMSO for 1 h at 37 °C, the capability of **L1** and **L2** to coordinate Pd was confirmed by ^1^H NMR. **L1-Pd** showed a clear shift of the aromatic protons from 6.7 and 6.6 ppm to 6.3 and 6.2 ppm. Additionally, phenolic protons (8.8 ppm) disappeared, corroborating the palladium-catecholate complexation (Figure 1a). ^1^H NMR spectrum of **L2-Pd** also showed a shift of the aromatic protons from 7.7, 7.5, and 7.0 ppm to 7.6, 7.4, and 6.6 ppm, corresponding to the enol form of methyl salicylate after complexation with Pd^57^ (Figure 1a; see Supporting Information Figure S1). The poor coordination yield of **L2-Pd** (10%) can be explained by having only one phenolic group donating electrons for complexation, while in the case of the catechol two phenolic electrons complete the coordination. Complete complexation was observed for the peptide ligands with Pd(II) (1 mol of Pd per mol of peptide; see ICP-OES, Figure 1f), possibly because of electron-donor groups from amino acid residues (see Supporting Information, Figures S7 and S8). To confirm the redox reaction, the UV–vis spectrum of **L1** and **L2** complexed with Pd was measured, displaying an absorbance increment at 300 nm compared to the source Pd(OAc)_2_ (Figure 1b). Based on these results, we decided to explore both ligands as a binding site for palladium on a peptide.

The peptide LLEYLKR was rationally selected from a pool of ribosomal peptides for being soluble in water (cLogP = −4.37) and for presenting hydrophobic (Leu, Tyr), acid (Glu), and basic (Lys, Arg) amino acid residues which have been reported as participating in catalytic mechanisms.^58^ The LLEYLKR peptide was synthesized on a Wang resin using standard Fmoc SPPS with HBTU/DIPEA as the coupling combination. 4-{(4-Hydroxy-3-methoxycarbonyl)phenyl]amino}-4-oxobutanoic acid (**4**) and 3,4-dihydroxyhydrocinnamic acid were coupled to the amino terminus of the peptide using HBTU/DIPEA.

The peptides were cleaved from the resin by treatment with TFA (5% DCM) and incubated in the presence of Pd(OAc)_2_ to form the metallopeptides **1-Pd** and **2-Pd** (Figure 1c). Both metallopeptides were purified by C18 SPE cartridges and characterized by mass spectrometry and ICP-OES (Figure 1d–f). Pd-peptides **1-Pd** and **2-Pd** showed relatively similar palladium mol % content per metallopeptide (52% and 51%, respectively); therefore full complexation was achieved (see full characterization in Supporting Information, Figures S6–S9 and Table S1). Importantly, mass spectra showed the remaining peak of the peptides **1** and **2**, which dissociate under electrospray ionization analysis conditions (see Figure 1d).

### Catalytic Studies

**Figure 2:**
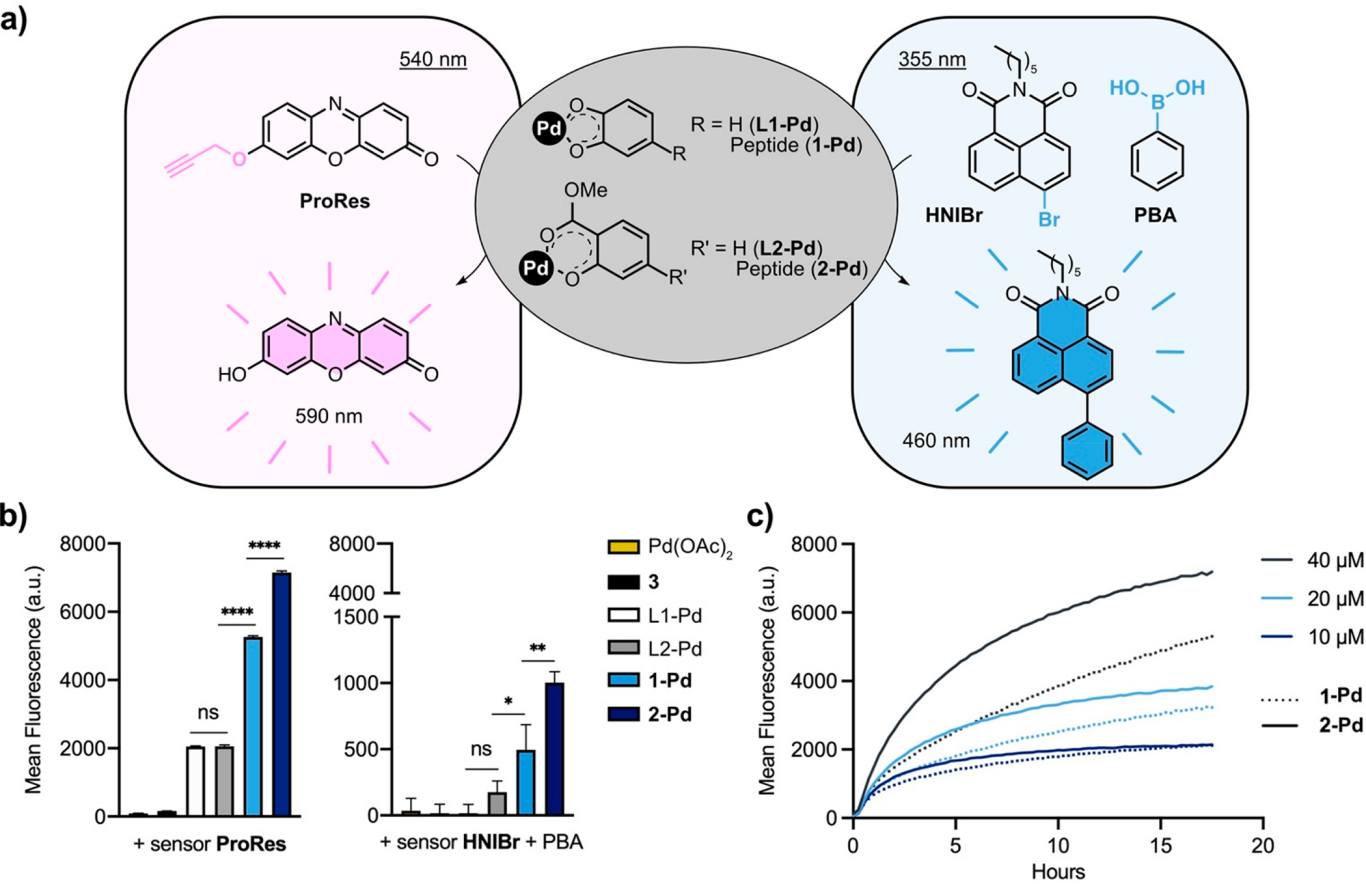
(a) Catalytic scheme of a depropargylation and SMCC reaction by Pd-peptide and Pd-phenolic complexes. (b) Conversion of an off-on sensor (*O*-propargyl-resorufin, **ProRes**) and two nonfluorescence building blocks (**HNIBr** and PBA) to the red fluorescent resorufin and the blue fluorescent naphthalimide derivative by the metallopeptides **1-Pd** and **2-Pd**. The catalytic efficiency after 18 h of incubation of the off–on sensor (**ProRes**, 40 μM or **HNIBr** + PBA, 100 μM) under physiological conditions (PBS, 37 °C, pH 7.4) with desalted catalysts (**1-Pd** and **2-Pd**). Pd concentrations for depropargylation were 5 and 6 μM, respectively, and for SMCC, they were 50 and 60 μM, respectively. Controls (desalted): Pd(OAc)_2_, peptide **3** (see Supporting Information for structure), **L1-Pd** and **L2-Pd** (10 μM for depropargylation and 100 μM for SMCC). Error bars: ±SD from *n* = 3. Significance was determined by one-way analysis of variance (ANOVA): ns (not significant, *P* > 0.5); *P < 0.05; **P < 0.005; ****P < 0.0001. (c) Kinetic study of the reaction of metallopeptides **1-Pd** and **2-Pd** (Pd concentrations of 5 and 6 μM, respectively) with different concentrations of an off–on sensor (**ProRes**, 40, 20, 10 μM) in PBS at 37 °C. The fluorescence was monitored every 15 min for 18 h. Curves were fitted using a nonlinear exponential equation.

To evaluate the catalytic activity of the metallopeptides, an off–on sensor (*O*-propargyl-resorufin, **ProRes**, 40 μM) was treated with metallopeptides **1-Pd** and **2-Pd** (Pd concentrations of 5 and 6 μM, respectively) in phosphate buffered saline (PBS, 37 °C, pH 7.4). Upon Pd catalyzed cleavage of the protecting group, fluorescence of resorufin was detected at 590 nm, showing 2.5-fold and 3.5-fold higher catalytic efficiency for metallopeptides **1-Pd** and **2-Pd** compared to **L1-Pd** and **L2-Pd** complexes, respectively (Figure 2a,b). To also investigate the catalytic ability of the metallopeptides to mediate the Suzuki–Miyaura cross-coupling reaction, a fluorescence-based catalytic study was performed. The chemosensor 4-bromo-*N*-*n*-butyl-1,8-naphthalimide^59^ (**HNIBr**, 100 μM) was incubated in the presence of phenylboronic acid (PBA, 100 μM) and Pd-peptides **1-Pd** and **2-Pd** (Pd concentrations of 50 and 60 μM, respectively) in PBS (37 °C, pH 7.4). Fluorescence signal of *N*-*n*-hexyl-4-phenyl-1,8-naphthalimide was detected at 460 nm upon ligation of previous building blocks by Pd-peptides **1-Pd** and **2-Pd**, while no fluorescence increase was shown for **L1-Pd** and **L2-Pd** complexes (Figure 2a,b). These results agree with previously reported catalytic studies on peptides, suggesting that amino acid residues must play a role in the mechanism of catalysis.^58,60^ But also, it is possible that higher concentration of hydrophobic substrate (e.g., **ProRes**) in hydrophobic pockets that might be formed by the peptide could accelerate the reaction rate.^40^ Importantly, Suzuki–Miyaura cross-coupling reaction showed lower catalytic efficiency than the *O*-depropargylation catalysis. This observation can be explained by an *in situ*, yet incomplete, reduction of Pd(II) to Pd(0), which is the active catalyst for Suzuki–Miyaura cross-coupling reaction.^61^

To further study the catalysis kinetics, Pd-peptides were then incubated with the nonfluorescent compound **ProRes** at different concentrations (40, 20, 10 μM), and fluorescence signal was monitored every 15 min over an 18 h period. As shown in the Figure 2c, the rate of product formation follows an exponential curve. Kinetic parameters were determined by plotting the Napierian logarithm of substrate **ProRes** concentrations versus time (see Supporting Information, Figure S10). Both metallopeptides **1-Pd** and **2-Pd** displayed pseudo-first-order kinetics (**1-Pd**, *K* = 0.1147 ± 0.05 and **2-Pd**, *K* = 0.2175 ± 0.03 h^–1^) and a half-life of 6.04 and 3.19 h, respectively. Therefore, we decided to do further biological studies with the metallopeptide **2-Pd**, having a depropargylation rate similar to previously reported bioorthogonal metal catalysts.^4,16,17,20^ Pd(II) complexes are rapidly deactivated by proteins;^38^ therefore the catalysis of **ProRes** by Pd-peptide (**2-Pd**) was tested in the presence of serum (see Supporting Information, Figure S11a). **2-Pd** remained functional, while the free Pd salt lost its catalytic activity in serum, confirming the protective role of the peptide scaffold.

### Cell Assays: Biocompatibility and *in Situ* Drug Synthesis

Paclitaxel (**PTX**) is an extremely potent microtubule inhibitor recommended for the treatment of the most common cancers, including breast, lung, and ovarian cancer.^62^ Despite all the severe side effects, myelosuppression, peripheral neuropathy, and cardiac toxicity, paclitaxel is currently enrolled in more than 1000 clinical trials. There is a huge unmet clinical need for a paclitaxel prodrug that could be applied globally, but only activated locally, to avoid these terrible off-target effects. A propargylated paclitaxel prodrug (**ProPTX**) stable in cell culture and uncaged by heterogeneous palladium catalysts has been reported.^20^ To further challenge the metallopeptide **2-Pd**, it was essential to determine its capability to catalyze the activation of paclitaxel by **ProPTX** depropargylation. Under physiological conditions (37 °C, PBS, pH 7.4) and in the presence of the metallopeptide **2-Pd**, **ProPTX** was converted into the cytotoxic agent **PTX** and detected by LCMS (see Supporting Information, Figure S12).

In parallel, we decided to test the versatility of the catalyst to synthesize the anticancer drug linifanib (**LNF**), an inhibitor of receptor tyrosine kinases, via Suzuki–Miyaura cross-coupling chemistry. The synthetic route of LNF includes a Suzuki–Miyaura cross-coupling of two building blocks (4-chloro-1*H*-indazol-3-amine (**A**) and 1-(2-fluoro-5-methylphenyl)-3-(4-(4,4,5,5-tetramethyl-1,3,2-dioxaborolan-2-yl)phenyl)urea (**B**)).^63^ Both building blocks **A** and **B** were incubated at 37 °C in the presence of the metallopeptide **2-Pd** (pH 7.4 in PBS) followed by the detection of the drug **LNF** by LCMS (see Supporting Information, Figure S13).

**Figure 3:**
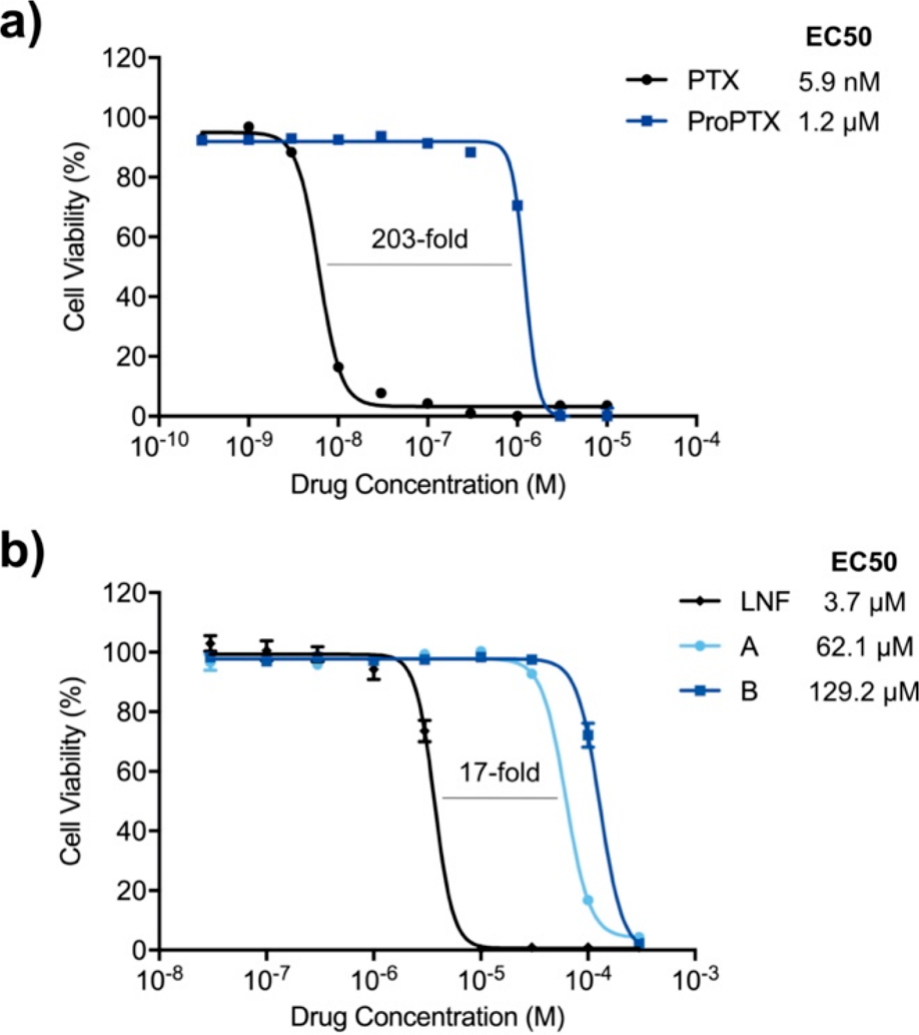
(a) Semilog dose–response curves and calculated EC_50_ values for A549 lung cancer cells after 5 days of treatment with **PTX** and **ProPTX** (0.03 nM to 10 μM). (b) Semilog dose–response curves and calculated EC_50_ values for A549 lung cancer cells after 5 days of treatment with **LNF**, **A**, and **B** (3 nM to 300 μM). Cell viability was measured at day 5. Error bars: ±SD from *n* = 3.

Motivated by these results, we sought to prove the anticancer effect of both drugs (**PTX** and **LNF**). Cells were treated with **PTX** and **LNF** at a range of concentrations (up to 300 μM), and cell viability measurements were carried out after 5 days of treatment. As expected, **PTX** induced a very potent cytotoxic effect (EC_50_ = 5.9 nM, Figure 3a) and **LNF** showed much lower activity (EC_50_ = 3.7 μM, Figure 3b). We then aimed to confirm the innocuous effect of the individual components involved in the catalysis. First, the dose–response curves for the **ProPTX** prodrug and the two building blocks (**A** and **B**) were represented and their EC_50_ values were calculated (EC_50_ of **ProPTX**, 1.2 μM; **A**, 62.1 μM; **B**, 129.2 μM). As expected, **ProPTX** displayed >200-fold lower activity than **PTX**;^20^ however the two building blocks (**A** and **B**) showed only >15-fold shift in the **LNF** apoptotic activity. The small gap between **LNF** and the two building blocks is mainly due to the low potency of **LNF** as a cytotoxic agent.

**Figure 4:**
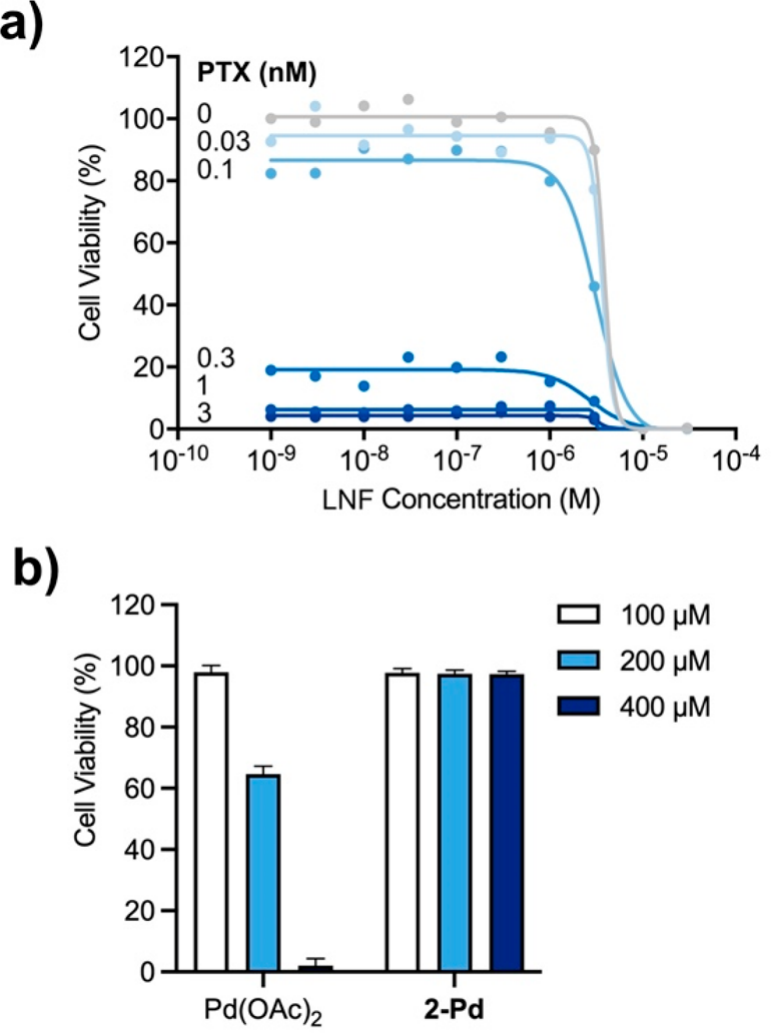
(a) Semilog dose–response curves for A549 lung cancer cells after 5 days of treatment with **LNF** (1 nM to 30 μM) in combination with **PTX** at increasing concentrations from 0 to 3 nM. (b) A549 cell viability study after treatment with Pd(OAc)_2_ and metallopeptide **2-Pd** at different concentrations (100–400 μM). Cell viability was measured at day 5. Error bars: ±SD from *n* = 3.

Next, a cell-based assay was performed to determine whether **PTX** and **LNF** show a synergic effect in non-small-cell lung cancer (NSCLC). A clinical study of paclitaxel in combination therapy with linifanib showed a reduced risk of progression or death in patients with NSCLC.^64^ Cancer cells were treated with **LNF** at different concentrations (1 nM to 30 μM) in combination with a range of nontoxic concentrations of **PTX** (0–3 nM). These results demonstrated the synergic effect between **LNF** and **PTX**, flattening the dose–response curves of **LNF** and showing 80% cell death at only 0.3 nM **PTX** (Figure 4a). In order to confirm that dual-synthesis of drugs can be performed and building blocks **A** + **B** do not help catalytic depropargylation, e.g., by forming hydrophobic interiors, the deprotection of the sensor (**ProRes**) was tested with **2-Pd** (Pd concentration of 6 μM) in the presence of **A** + **B** (2.5 or 25 μM, PBS, pH 7.4). The catalytic efficiency decreases as more **A** + **B** was added (see Supporting Information, Figure S11b), corroborating that any improvement in the therapeutic effect by dual-synthesis of drugs must be synergistic.

In parallel, to confirm the lack of toxicity of the catalyst, we performed a cell viability assay to evaluate the toxicity of the metallopeptides in A549 lung cancer cells. As shown in Figure 4b, Pd(OAc)_2_ displayed toxicity at 200 μM while metallopeptide **2-Pd** did not manifest any cytotoxic effect at the concentrations tested (up to 400 μM).

**Figure 5:**
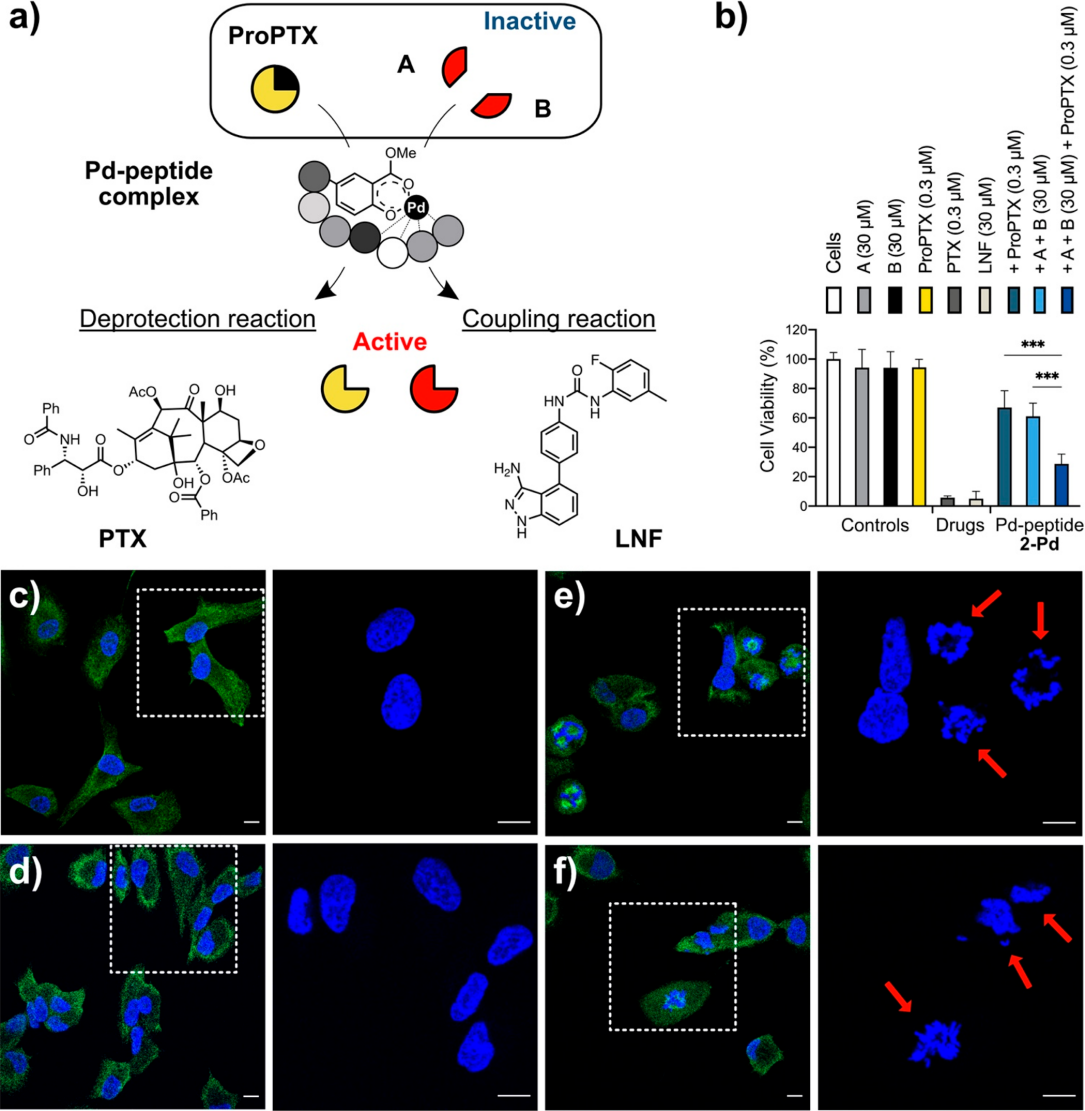
Metallopeptide **2-Pd** catalyzed activation and synthesis of two anticancer drugs: **PTX** and **LNF**. (a) Simultaneous depropargylation of **ProPTX** and Suzuki–Miyaura coupling of **A** and **B**. (b) Cell viability assay of A549 lung cancer cells treated with **2-Pd** (160 μg/mL, Pd concentration of 75 μM) in combination with **ProPTX** (0.3 μM) or/and **A** + **B** (30 μM) for 5 days (PrestoBlue assay *n* = 3). Significance was determined by one-way analysis of variance (ANOVA): ***P < 0.001. (c–f) Immunofluorescence study with FITC/DAPI channels merged (left) and the DAPI channel expanded (right) to clearly show nuclear damage: (c) control; (d) 0.3 μM **ProPTX** + 30 μM **A** + **B**; (e) 0.3 μM **PTX** + 30 μM **LNF**; (f) 160 μg/mL metallopeptide **2-Pd** (Pd concentration of 75 μM) + 0.3 μM **ProPTX** + 30 μM **A** + **B** (drug synthesis experiments). 24 h after treatment, cells were fixed and stained with anti-α-tubulin IgG (green) and DAPI (blue). Scale bar = 10 μm.

The dual prodrug activation/drug synthesis was evaluated in a cell-based study using metallopeptide **2-Pd** (160 μg/mL, Pd concentration of 75 μM) (Figure 5a). A549 lung cancer cells were incubated with either **ProPTX** (0.3 μM), precursors **A** and **B** (30 μM), or a mixture of **ProPTX**, **A**, and **B** in the presence of **2-Pd**. Control cells incubated only with metallopeptide **2-Pd**, individual precursors, or prodrug did not show any cell death (see Supporting Information, Figure S14). When combining the catalysts and the precursors, cell viability decreased to 67% with prodrug **ProPTX** only and to 61% when incubated with building blocks **A** and **B**. In contrast, simultaneous treatment (incubation with **ProPTX**, **A**, and **B**) caused cell viability to decrease to 28% after 5 days (Figure 5b). These results confirm the synergic effect when combining the deprotection and cross-coupling chemistry. Pd(II) deactivation by serum is a well reported issue and we can see this phenomenon occurring readily with Pd(OAc)_2_ (see Supporting Information Figure S14). Importantly, the catalyst **2-Pd** remains active in the presence of serum.

Finally, to validate that the combined treatment of Pd-peptide and drug precursors results in the same antiproliferative mode of action than the parent drugs **PTX** and **LNF**, we studied microtubule and nucleus stabilization by immunofluorescence.^20^ Cells were fixed 24 h after treatment, incubated with cell nuclei DAPI stain and anti-α-tubulin IgG, and imaged by confocal microscopy. As shown in Figure 5c,d, negative controls did not induce changes in cell morphology (see controls with individual components in Supporting Information, Figures S15 and S16). In contrast, treatment of A549 cells with **PTX** + **LNF** led to round-shape morphology with nuclear fragmentation and microtubule condensation (Figure 5e). Importantly, equivalent morphological changes were observed in cells treated with the Pd-peptide and drug precursors combination, evidence that the anticancer effect mediated by the combination treatment is the result of *in situ* drug generation (Figure 5f).

## Conclusions

A methyl salicylate peptide LLEYLKR (metallopeptide **2-Pd**) efficiently forms a palladium complex and displays bioorthogonal catalytic properties in the presence of cultured lung cancer cells. Metallopeptide **2-Pd** did not show any cytotoxic effect on its own, confirming the crucial role of the peptide to limit metal toxicity. Additionally, the peptide improved the catalytic efficiency of palladium, demonstrating its contribution to the mechanism of catalysis. The versatility of the catalyst in biological environments was exemplified by mediating two types of chemistry in the presence of cultured human cells: a propargyl deprotection and a Suzuki–Miyaura cross-coupling reaction. These can be executed in parallel, leading to a synergic effect of the two anticancer drugs by the simultaneous catalytic synthesis of paclitaxel and linifanib in human lung cancer cells. This is the first demonstration of multiple reactions being catalyzed in parallel by a homogeneous catalyst for drug synthesis. This opens the possibility of more advanced drug combination therapies with increased efficacy and reduced side effects and improved cancer targeting by the catalyst. Novel bioorthogonal homogeneous catalysts, as presented here, further facilitate the possibility of targeted catalysis by direct coupling to delivery vehicles such as antibodies, to overcome the current challenge of delivering the catalyst inside the human body to the desired location of action.

## Experimental Section

### General

Chemicals and solvents were purchased from Sigma-Aldrich, abcr Germany, Axon Medchem, ChemPUR. FmocArg(Pbf)OH, FmocLys(Boc)OH, FmocLeuOH, FmocTyr(tBu)OH, FmocGlu(tBu)OH were purchased from GL Biochem. All commercial amino acids are optically pure l-enantiomers. NMR spectra were recorded at ambient temperature on a 500 MHz Bruker Avance III spectrometer. Chemical shifts are reported in parts per million (ppm) relative to the solvent peak. Rf values were determined on Merck TLC silica gel 60 F254 plates under a 254 nm UV source. Purifications were carried out by Biotage Selekt flash column chromatography system or via semipreparative TLC chromatography on Merck TLC silica gel 60 F254 plates. All compounds are >95% pure as measured by either HPLC and NMR or HPLC. HPLC was performed on a Shimadzu LC-20AD system with a ReproSil-XR 120 C18, length 150 mm, i.d. 4.6 mm, 3 μm and coupled to a SPD-M20A diode array detector. The following eluents were used: (A) H_2_O + 0.1% TFA; (B) AcN. Method: (0.5 min) 80% (A) to 10% (A) in (B) over 10 min, then 10% (A) in (B) over 2 min, then 10% (A) in (B) to 80% (A) over 1 min, then 80% (A) over 5 min (flow 1 mL/min). LCMS was performed on an Agilent 1200 Chemstation analytical system with a Grom-Sil-120-ODS-4-HE (Grace), length 50 mm, i.d. 2 mm, 3 μm and coupled directly to a LTQ Orbitrap XL mass spectrometer (Thermo Fisher Scientific, ion source ESI). The following eluents were used: (A) H_2_O + 0.1% FA; (B) AcN + 0.1% FA. Method: (1 min) 95% (A) to 5% (A) in (B) over 10 min, then 5% (A) in (B) over 1 min, then 5% (A) in (B) to 95% (A) over 5 min, then 95% (A) over 1 min (flow 0.3 mL/min). ICP-OES measurements were carried out in a Varian 715 ICP optical emission spectrometer, with samples at 20 mg/L in 10% HNO_3_ in water. Stock solutions (100 mM) were prepared in DMSO.

### Synthesis of Compounds

5-Aminosalicylic acid methyl ester,^65^*O*-propargyl-resorufin (**ProRes**),^17^ and 6-bromo-2-hexyl-1*H*-benzo[*de*]isoquinoline-1,3(2*H*)-dione (**HNIBr**),^66^ 1-(2-fluoro-5-methylphenyl)-3-(4-(4,4,5,5-tetramethyl-1,3,2-dioxaborolan-2-yl) phenyl)urea (**B**)^63^ and **ProPTX**(20) were synthesized according to previously reported procedures. The spectral data matched the values reported in the literature, and all compounds were >95% pure by HPLC and ^1^H NMR analysis.

### Synthesis of Peptide H-Leu-Leu-Glu-Tyr-Leu-Lys-Arg-OH (**3**)

Wang resin (0.22 g, 0.92 mmol/g) was swollen in 5 mL of DMF for 30 min. Fmoc-Arg(Pbf)-OH (10 equiv) was dissolved in dry DCM, and a solution of DIC (5 equiv) in dry DCM was added. The mixture was stirred for 20 min at 0 °C, followed by removal of the DCM in vacuo. The residue was dissolved in 5 mL of DMF and the solution added to the swollen resin. DMAP (0.1 equiv) in 1 mL of DMF was added to the resin mixture, which was agitated for 2 h. The resin was then filtered and washed with DMF (×3), DCM (×3), MeOH (×3), Et_2_O (×2) and dried under vacuum for 30 min and the level of attachment estimated using the quantitative Fmoc test.^67^ Fmoc removal was performed using 20% piperidine in DMF for 20 min (×2). The resin was then filtered and washed with DMF (×3), DCM (×3), MeOH (×3). Resin was swollen in 5 mL of DCM. *N*-Fmoc-amino acid (5 equiv) and HBTU (4.9 equiv) were dissolved in DMF (0.1 M). DIPEA (10 equiv) was added, and the resulting mixture was added to the resin. The resin was agitated for 40 min. The resin was washed with DMF (×3), DCM (×3), MeOH (×3), Et_2_O (×2). The completion of each coupling was verified using the qualitative ninhydrin test.^68^

*Cleavage of Peptides from Resin (50 mg, 0.67 mmol/g):* TFA/TIS/H_2_O (95/2.5/2.5) was added and the resin stirred for 2 h. The TFA solution was removed, concentrated to 1 mL, and added to cold Et_2_O in a centrifuge tube. The resulting precipitate was collected by centrifugation, washed with Et_2_O (×4) and lyophilized to afford **3** as a white solid (30 mg, 95%). *m*/*z* (ES+): 934.5720 (M + H)^+^, 467.7898 (M + 2H)^2+^, 312.1956 (M + 3H)^3+^ (100%). HRMS (ES+) for C_44_H_76_N_11_O_11_ (M + H)^+^: calcd 934.5720, found 934.5720; purity as measured by HPLC was >99%.

### Synthesis of 4-{(4-Hydroxy-3-(methoxycarbonyl)phenyl]amino}-4-oxobutanoic Acid (**4**)

5-Aminosalicylic acid methyl ester (0.42 g, 2.5 mmol) in DCM (2.5 mL) was added to succinic anhydride (0.23 g, 2.25 mmol) in DCM (2.5 mL). The reaction mixture was irradiated at 100 °C for 10 min and the resulting solid was filtered and recrystallized in MeOH to give the title compound as a pale-brown solid (0.57 g, 2.13 mmol, 94%). ^1^H NMR (400 MHz, DMSO-*d*_6_) δ 12.10 (s, 1H), 10.24 (s, 1H), 9.94 (s, 1H), 8.15 (d, *J* = 2.7 Hz, 1H), 7.61 (dd, *J* = 9.0, 2.7 Hz, 1H), 6.93 (d, *J* = 8.9 Hz, 1H), 3.89 (s, 3H), 2.52–2.48 (m, 4H); ^13^C NMR (101 MHz, DMSO-*d*_6_) δ 173.77, 169.80, 169.06, 155.76, 131.26, 127.10, 119.79, 117.48, 112.31, 52.44, 30.82, 28.78. *m*/*z* (ES−): 266.0 (M – H)^−^ (100%). HRMS (ES+) for C_12_H_14_NO_6_ (M + H)^+^: calcd 268.0816, found 268.0817; purity as measured by ^1^H and ^13^C NMR was >99%.

### Synthesis of 3-(3,4-Dihydroxyphenyl)propanamide-Leu-Leu-Glu-Try-Leu-Lys-Arg-OH (**1**)

3,4-Dihydroxyhydrocinnamic acid (0.22 mmol, 40 mg, 5 equiv) and HBTU (4.9 equiv) were dissolved in DMF (0.1 M). DIPEA (10 equiv) was added, and the resulting mixture was added to the resin previously synthesized (65 mg, 0.67 mmol/g). The resin was agitated for 40 min. The resin was washed with DMF (×3), DCM (×3), MeOH (×3), Et_2_O (×2). Coupling was confirmed using a qualitative ninhydrin test.^68^ Peptide **1** was cleaved using the previous cleavage of peptides from resin procedure and lyophilized to afford white solid (52 mg, 92%). *m*/*z* (ES+): 1098.6293 (M + H)^+^, 549.8135 (M + 2H)^2+^. HRMS (ES+) for C_53_H_84_N_11_O_14_ (M + H)^+^: calcd 1098.6199, found 1098.6193; purity as measured by HPLC was >95%.

### Synthesis of Methyl-2-hydroxy-5-(4-oxobutanamide)benzoate-Leu-Leu-Glu-Try-Leu-Lys-Arg-OH (**2**)

3-4-{[4-Hydroxy-3-(methoxycarbonyl)phenyl]amino}-4-oxobutanoic acid (0.22 mmol, 59 mg, 5 equiv) and HBTU (4.9 equiv) were dissolved in DMF (0.1 M). DIPEA (10 equiv) was added, and the resulting mixture was added to the resin previously synthesized (65 mg, 0.67 mmol/g). The resin was agitated for 40 min. The resin was washed with DMF (×3), DCM (×3), MeOH (×3), Et_2_O (×2). Coupling was confirmed using a qualitative ninhydrin test.^68^ Peptide **2** was cleaved using the previous cleavage of peptides from resin procedure and lyophilized to afford a white solid (50 mg, 96%). *m*/*z* (ES+): 1183.6354 (M + H)^+^, 592.3217 (M + 2H)^2+^. HRMS (ES+) for C_56_H_87_N_12_O_16_ (M + H)^+^: calcd 1183.6358, found 1183.6354; purity as measured by HPLC was >98%.

### Synthesis of Metallopeptide **1-Pd**

Peptide **1** (100 μM) was incubated in the presence of Pd(OAc)_2_ (50, 100, 200, 400 μM) in 0.5% DMSO/PBS (0.5 mL) at 37 °C, 1200 rpm in a Thermomixer for 2 h. The mixture was purified using a C18 reverse phase cartridge to remove the excess of Pd(OAc)_2_ and analyzed by MS and ICP-OES. HRMS (ES+) for metallopeptide **1-Pd** C_53_H_83_N_11_O_14_Pd (M + 2H)^2+^: calcd 601.7572, found 601.7567; ICP-OES 52.41 ± 5.71 nmol Pd (molar ratio Pd:peptide 1:1).

### Synthesis of Metallopeptide **2-Pd**

Peptide **2** (100 μM) was incubated in the presence of Pd(OAc)_2_ (50, 100, 200, 400 μM) in 0.5% DMSO/PBS (0.5 mL) at 37 °C, 1200 rpm in a Thermomixer for 2 h. The mixture was purified using a C18 reverse phase cartridge to remove the excess of Pd(OAc)_2_ and analyzed by MS and ICP-OES. HRMS (ES+) for metallopeptide **2-Pd** C_56_H_86_N_12_O_16_Pd (M + 2H)^2+^: calcd 644.2654, found 644.2650; ICP-OES 51.65 ± 3.92 nmol Pd (molar ratio Pd:peptide 1:1).

### Pd Complexation Studies

The ligands (1,2-dihydroxybenzene (**L1**); methyl salicylate (**L2**); peptide **2**) (50 mM) were dissolved in the presence of Pd(OAc)_2_ (50 mM) in DMSO-*d*_6_ (0.5 mL) and incubated at 37 °C for 1 h. The mixtures were analyzed by ^1^H NMR.

### UV–Visible Studies

The ligands (1,2-dihydroxybenzene (**L1**); methyl salicylate (**L2**); peptide **1**; peptide **2**) (100 μM) were incubated in the presence of Pd(OAc)_2_ (100 μM) in PBS (1 mL) at 37 °C for 2 h, 1200 rpm in the Thermomixer. The mixture was purified using a C18 reverse phase cartridge to remove the excess of Pd(OAc)_2_ and redissolved in PBS at 100 μM. The UV–visible spectrum was measured in a FLUOstar Omega multimode reader.

### Fluorogenic Assay of Depropargylations

**ProRes** (10, 20, and 40 μM) was dissolved in a PBS or 10% FBS/PBS solution (200 μL) with metallopeptides **1-Pd** and **2-Pd** (Pd concentrations of 5 and 6 μM, respectively) in triplicates. As control Pd(OAc)_2_ or desalted Pd(OAc)_2_, peptide **3**, **L1-Pd**, and **L2-Pd** were used at 10 μM. In parallel, monomers **A** + **B** (2.5 or 25 μM) were tested in combination with **2-Pd** (Pd concentration of 6 μM). The mixtures were shaken at 1200 rpm and 37 °C in a Thermomixer for 18 h. Reaction crudes were transferred to a 96-well plate format and were measured in a FLUOstar Omega multimode reader (Ex/Em: 540 nm/590 nm). For the kinetic studies, the mixtures were shaken at 37 °C and monitored over time (every 15 min, 18 h) by fluorescence in a FLUOstar Omega multimode reader (Ex/Em: 540 nm/590 nm).

### Suzuki–Myaura Cross-Coupling Screening

**HNIBr** (100 μM) and phenylboronic acid (PBA, 100 μM) were dissolved in a PBS solution (200 μL) with metallopeptides **1-Pd** and **2-Pd** (Pd concentration of 50 and 60 μM, respectively) in triplicates. As control desalted Pd(OAc)_2_, **3**, **L1-Pd**, and **L2-Pd** were used at 100 μM. The mixtures were shaken at 1200 rpm and 37 °C in a Thermomixer for 18 h. Reaction crudes were transferred to a 96-well plate format and were measured in a FLUOstar Omega multimode reader (Ex/Em: 355 nm/460 nm).

### Synthesis of Drugs Paclitaxel (PTX) and Linifanib (LNF) by **2-Pd**

**ProPTX** (100 μM) or **A** and **B** (100 μM, each) were dissolved in a PBS solution (200 μL) with metallopeptide **2-Pd** (Pd concentration of 6 μM), respectively. The mixtures were shaken at 1200 rpm and 37 °C in a Thermomixer for 24 h. Samples were desalted by StageTips and analyzed by LCMS (Agilent 1200) using an Orbitrap XL mass spectrometer (Thermo Fisher, Ion source ESI). **PTX**, **ProPTX**, **LNF**, **A**, and **B** (100 μM, each) in PBS were used as analytical controls.

### Cell Culture

Human lung adenocarcinoma A549 cells were cultured in Dulbecco’s modified Eagle medium (DMEM) supplemented with serum (10% FBS) and l-glutamine (2 mM) and incubated in a tissue culture incubator at 37 °C and 5% CO_2_. The growth medium was removed prior to the addition of the metallopeptides and inactive constituents, which were dissolved in fresh growth medium supplemented with 10% FBS.

### Biocompatibility Assays

Biocompatibility of metallopeptides was compared by performing dose–response studies in A549 cells. Cells were seeded in a 96-well plate format (at 1500 cells/well) and incubated for 48 h before treatment. Each well was then replaced with fresh medium (supplemented with 10% FBS) containing metallopeptides or Pd(OAc)_2_ (100, 200, 400 μM) and incubated for 5 d. Untreated cells were incubated with DMSO (0.1% v/v). Experiments were performed in triplicate. PrestoBlue cell viability reagent (10% v/v) was added to each well and the plate incubated for 60 min. Fluorescence emission was detected using a FLUOstar Omega multimode reader (excitation filter at 540 nm and emissions filter at 590 nm). All conditions were normalized to the untreated cells (100%).

### Dose–Response Curves of Active and Inactive Agents

The antiproliferative activities of **PTX**/**ProPTX** and **LNF**/**A**/**B** were compared by performing dose–response studies against the A549 cells. Cells were seeded in a 96-well plate format (at 1500 cells/well) and incubated for 48 h before treatment. Each well was then replaced with fresh medium (supplemented with 10% FBS) containing **PTX**/**ProPTX** (0.03 nM to 10 μM) or **LNF**/**A**/**B** (3 nM to 300 μM). Untreated cells were incubated with DMSO (0.1% v/v). After 5 d of incubation, cell viability was determined as described above. All conditions were normalized to the untreated cells (100%) and curves fitted using GraphPad Prism using a sigmoidal variable slope curve. Experiments were performed in triplicate.

### Combination Therapy Assay

The antiproliferative activities of **PTX** and **LNF** combination treatment was done by performing dose–response studies against the A549 cells. Cells were seeded in a 96-well plate format (at 1500 cells/well) and incubated for 48 h before treatment. Each well was then replaced with fresh medium (supplemented with 10% FBS) containing **PTX** (0.03–3 nM) or/and **LNF** (0.001–30 μM). Untreated cells were incubated with DMSO (0.1% v/v). After 5 d of incubation, cell viability was determined as described above. All conditions were normalized to the untreated cells (100%) and curves fitted using GraphPad Prism using a sigmoidal variable slope curve. Experiments were performed in triplicate.

### Synthesis of Drugs by Metallopeptide **2-Pd**

A549 cells were plated as described above. Each well was then replaced with fresh medium (supplemented with 10% FBS) containing metallopeptide **2-Pd** (160 μg/mL, Pd concentration of 75 μM) or Pd(OAc)_2_ (40 μg/mL); **PTX** and **ProPTX** (0.3 μM, each); **LNF**, **A** and **B** (30 μM, respectively); or combination of metallopeptide **2-Pd** + **ProPTX** or **A** + **B** or **ProPTX** + **A** + **B** (**ProPTX** 0.3 μM, **A** and **B** 30 μM, respectively). All experiments, including the untreated cells, containing 0.1% v/v DMSO, were performed in triplicate. After 5 d of incubation, cell viability was determined as described above. All conditions were normalized to the untreated cells (100%).

### Immunofluorescence Assay

A549 cells were seeded on 18 mm poly(l-lysine)-precoated coverslips in 12-well plates (50 000 cells/well). Cells were incubated 24 h before treatment, and each well was replaced with fresh medium (supplemented with 10% FBS) containing: control, **ProPTX** (0.3 μM), **A** + **B** (30 μM), **ProPTX** (0.3 μM) + **A** + **B** (30 μM), **PTX** (0.3 μM) + **LNF** (30 μM), metallopeptide **2-Pd** (160 μg/mL, Pd concentration of 75 μM), metallopeptide **2-Pd** (160 μg/mL, Pd concentration of 75 μM) + **ProPTX** (0.3 μM) + **A** + **B** (30 μM). After 24 h, cells were fixed with paraformaldehyde (4% v/v) for 10 min and washed with PBS 3 times. Cells were permeabilized for 15 min in PBS, Tween (0.3% v/v) and washed 3 times with PBS. Coverslips were then covered with a blocking solution (PBS, 5% FBS, 0.3% Triton X-100) for 60 min. Cells were washed with PBS 3 times and incubated in an antibody dilution buffer (PBS, 1% BSA, 0.3% Triton X-100) containing anti-α-tubulin mAb Alexa Fluor 488 (Santa Cruz) at a dilution of 1:200, overnight at 4 °C. Coverslips were washed 3 times with PBS and mounted on Superfrost microscope slides (Thermo Fisher) with ProLong gold mounting medium with DAPI (Thermo Fisher). Cells were imaged using a scanning confocal inverted microscope Nikon scanning confocal A1Rsi+ with a 60× oil immersion objective. The images were acquired using the NIS-Elements program in a sequential mode and analyzed with ImageJ software to obtain maximal projections.

## Supporting Information Available

Methods; redox mechanism scheme, Figure S1; HRMS and HPLC data of peptides, Figures S2–S4; NMR spectra of compound **4**, Figure S5; HRMS, ICP-OES, NMR, and UV–vis spectra of metallopeptides, Figures S6–S9; determination of the reaction rate constant and half-life of metallopeptides, Figure S10; stability studies of the catalyst **2-Pd**, Figure S11; LCMS of the synthesized drugs paclitaxel (PTX) and linifanib (LNF) by metallopeptide **2-Pd**, Figures S12 and S13; cell viability and confocal images of the synthesized drugs by metallopeptide **2-Pd** in cell culture, Figures S14–S16 (PDF)
SMILES molecular formula strings (CSV)

## Abbreviations Used

AcN: acetonitrile
Arg: arginine
ArMs: artificial metalloenzymes
Boc: *tert*-butyloxycarbonyl
BSA: bovine serum albumin
d: day
DAPI: 4′,6-diamidino-2-phenylindole
DCM: dichloromethane
DIC: *N*,*N*′-diisopropylcarbodiimide
DIPEA: *N*,*N*-diisopropylethylamine
DMAP: 4-dimethylaminopyridine
DMEM: Dulbecco’s modified Eagle’s medium
DMF: dimethylformamide
DMSO: dimethyl sulfoxide
EC_50_: half-maximal effective concentration
equiv: equivalents
ES: electrospray
Et_2_O: diethyl ether
FA: formic acid
FBS: fetal bovine serum
Fmoc: fluorenylmethyloxycarbonyl
Glu: glutamic acid
h: hours
HBTU: (2-(1*H*-benzotriazol-1-yl)-1,1,3,3-tetramethyluronium hexafluorophosphated
HPLC: high-performance liquid chromatography
HRMS: high-resolution mass spectrometry
ICP-OES: inductively coupled plasma optical emission spectroscopy
IgG: immunoglobulin G
LCMS: liquid chromatography–mass spectrometry
Leu: leucine
Lys: lysine
MeOH: methanol
min: minutes
MS: mass spectrometry
NMR: nuclear magnetic resonance
NSCLC: non-small-cell lung carcinoma
ppm: parts per million
Pbf: 2,2,4,6,7-pentamethyldihydrobenzofuran-5-sulfonyl
PBS: phosphate buffered saline
Pd: palladium
Pd(OAc)_2_: palladium diacetate
Rf: retention factor
rpm: revolutions per minute
rt: room temperature
SD: standard deviation
SMCC: Suzuki−Miyaura cross-coupling
SPE: solid-phase extraction
SPPS: solid-phase peptide synthesis
tBu: *tert*-butyl
TFA: trifluoroacetic acid
TIS: triisopropylsilane
TLC: thin-layer chromatography
TMC: transition-metal catalysts
Tyr: tyrosine
UV–vis spectroscopy: ultraviolet–visible spectroscopy
