# Supplementary material for: Dual-Bioorthogonal Catalysis by a Palladium Peptide Complex
Source: J Med Chem. 2023 Feb 23;66(5):3301–11. doi: 10.1021/acs.jmedchem.2c01689 (PMC10009749; doi:10.1021/acs.jmedchem.2c01689)
Supplement: Supplementary file 1 — jm2c01689_si_001.pdf [file jm2c01689_si_001.pdf]

SUPPORTING INFORMATION

Dual-bioorthogonal catalysis by a palladium peptide complex

Ana M. Pérez-López,[1,2,‡] Adam Belsom,[1,2,‡] Linus Fiedler,[1,2] Xiaoyi Xin,[1,2] Juri Rappsilber[1,2,3,*]

1 Technische Universität Berlin, Chair of Bioanalytics, 10623 Berlin, Germany

2 Si-M/"Der Simulierte Mensch", a Science Framework of Technische Universität Berlin and Charité - Universitätsmedizin Berlin, 10623 Berlin, Germany.

3 Wellcome Centre for Cell Biology, University of Edinburgh, Edinburgh EH9 3BF, UK.

* juri.rappsilber@tu-berlin.de

Table of Contents

1. Redox mechanism S2
2. Characterization of the compounds S2
    2.1. Characterization of peptide **1** S2
    2.2. Characterization of peptide **2** S3
    2.3. Characterization of peptide **3** S3
    2.4. NMR of compound **4** S4
    2.5. Characterization of metallopeptides **1-Pd** and **2-Pd** S5
3. Determination of the reaction rate constant (K) and half-life ($t_{1/2}$) S7
4. Stability studies of the catalyst **2-Pd** S8
5. Synthesis of drugs paclitaxel and linifanib by metallopeptide **2-Pd** S9
6. Biological studies S10
    6.1. Synthesis of drugs by metallopeptide **2-Pd** S10
    6.2. Immunofluorescence assay S10

1. Redox mechanism

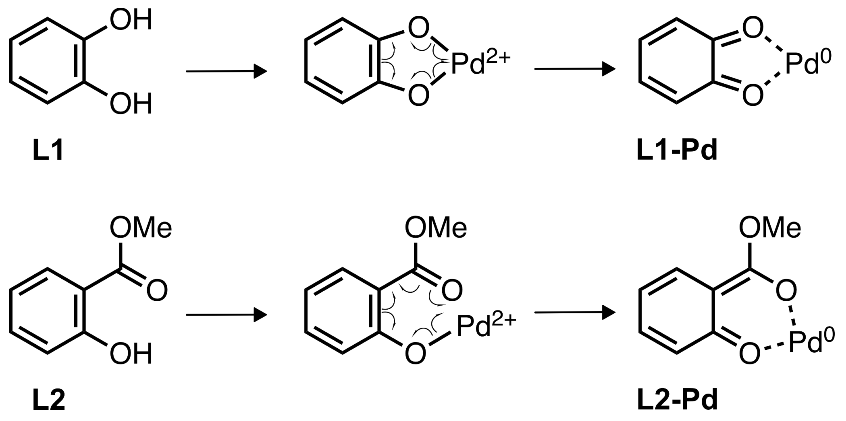

Figure S1. Proposed reduction mechanism steps of Pd by 1,2-dihydroxybenzene[1] (**L1**) and methyl salicylate (**L2**).

2. Characterization of compounds

2.1. Characterization of 3-(3,4-dihydroxyphenyl)propanamide Leu-Leu-Glu-Try-Leu-Lys-Arg-OH (1)

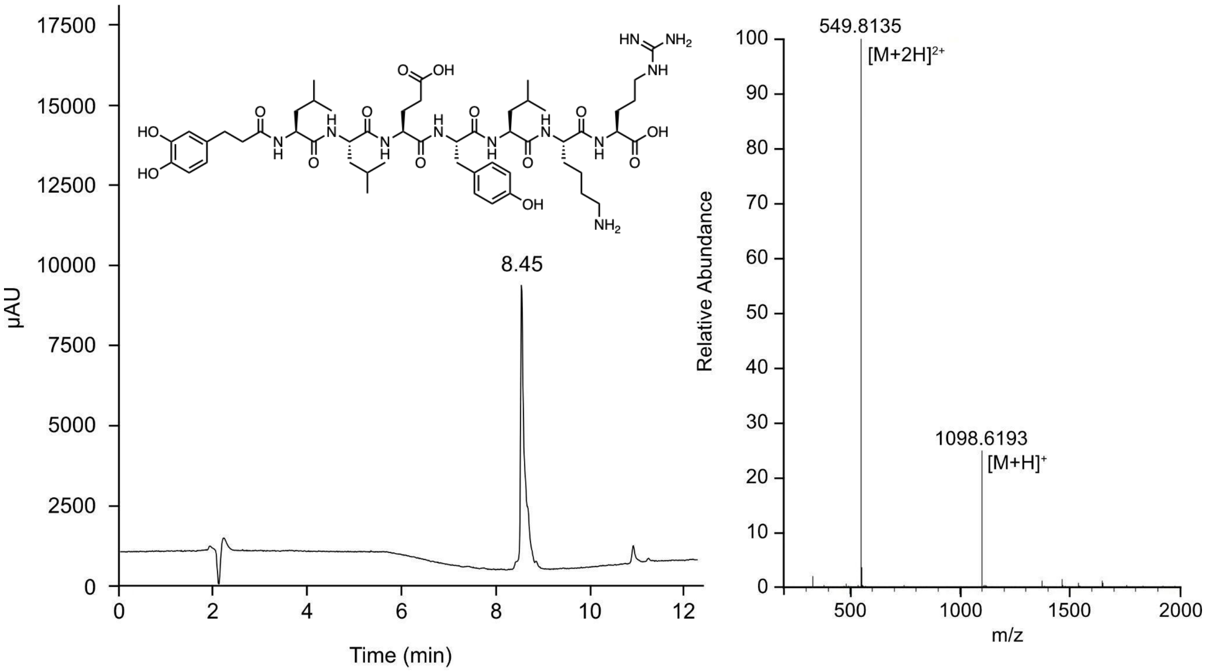

Figure S2. HPLC and MS of peptide **1** (RT = 8.45 min, purity 95.1% (254 nm)).

2.2. Characterization of methyl-2-hydroxy-5-(4-oxobutanamide)benzoate Leu-Leu-Glu-Try-Leu-Lys-Arg-OH (2)

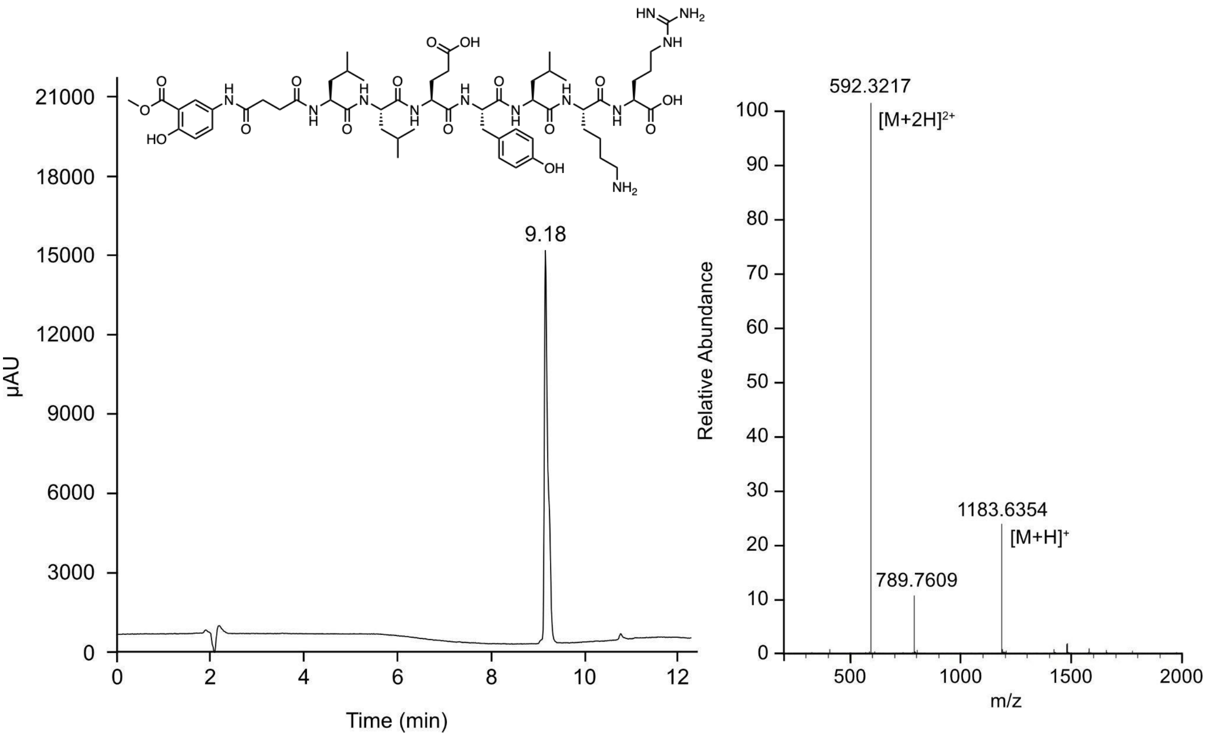

Figure S3. HPLC and MS of peptide **2** (RT = 9.18 min, purity 98.4% (254 nm)).

2.3. Characterization of peptide H-Leu-Leu-Glu-Tyr-Leu-Lys-Arg-OH (3)

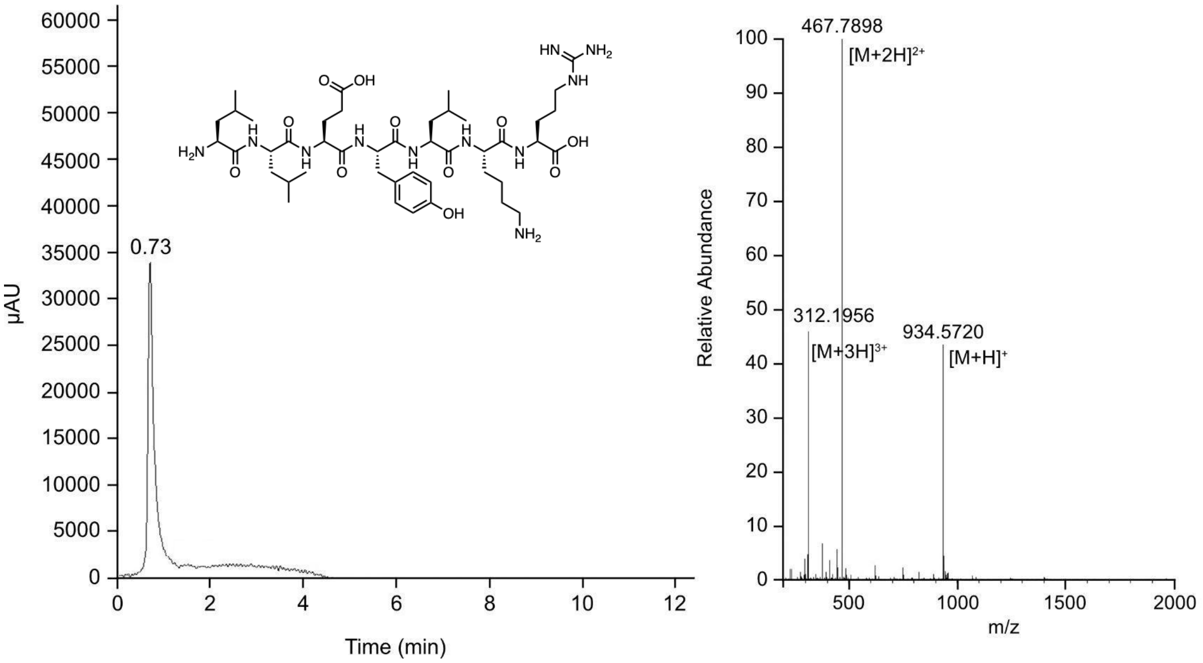

Figure S4. HPLC and MS of peptide **3** (RT = 0.73 min, purity 99.5% (254 nm)).

2.4. NMR of 4-{(4-hydroxy-3-(methoxycarbonyl)phenyl]amino}-4-oxobutanoic acid (4)

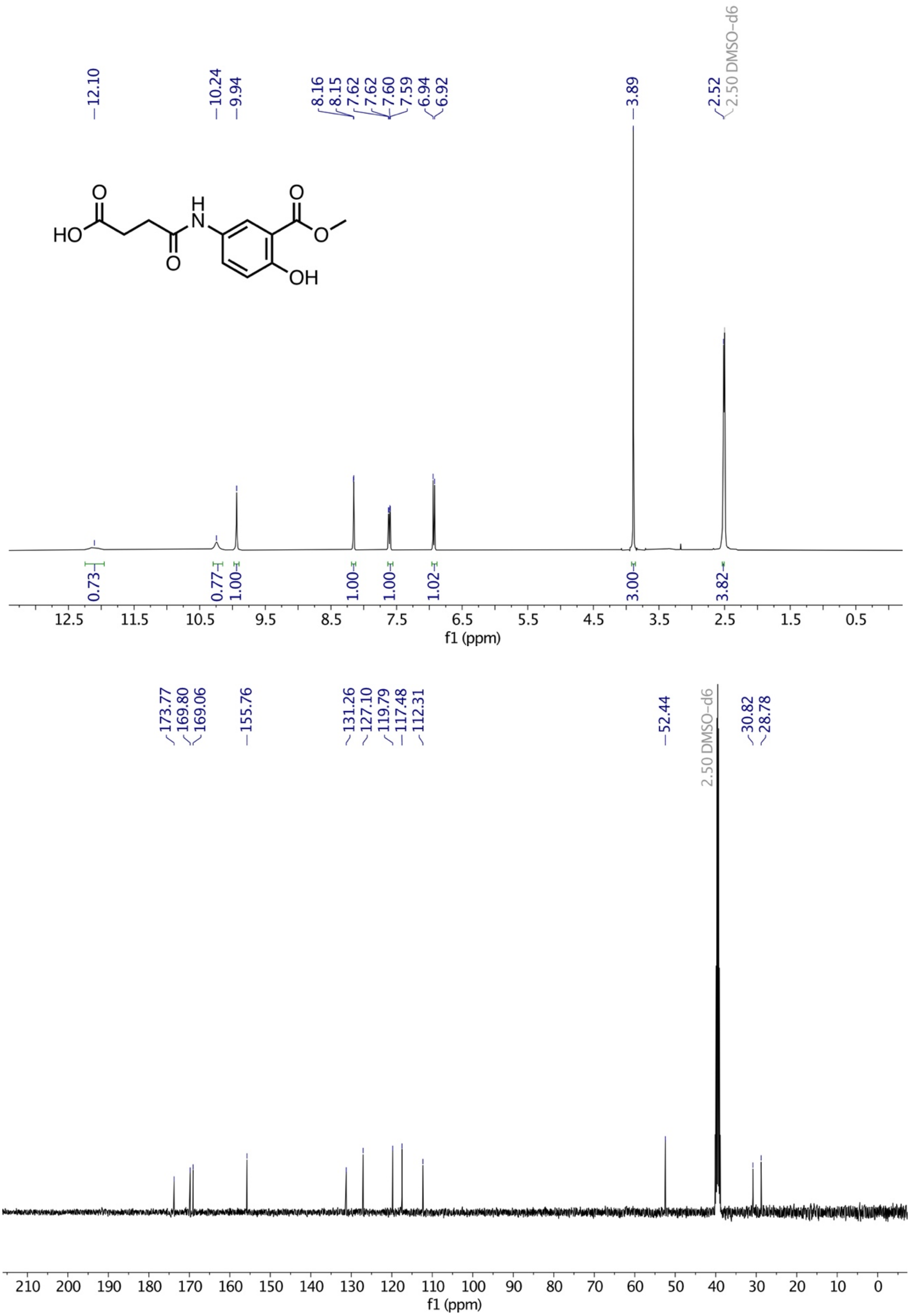

Figure S5. ^{1}H and ^{13}C-NMR of compound **4**.

2.5. Characterization of metallopeptides 1-Pd and 2-Pd

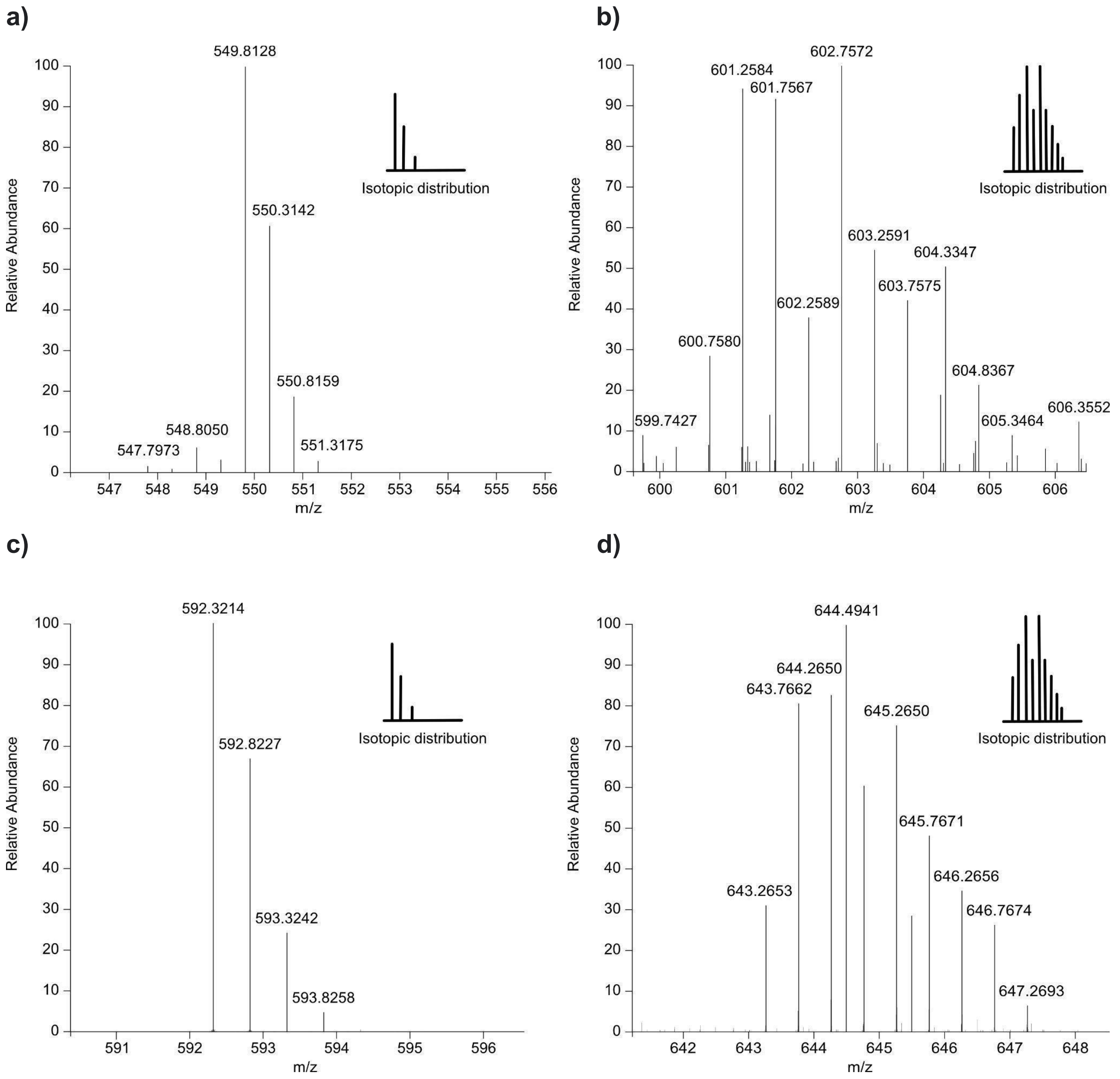

Figure S6. Isotopic distribution for the $[M+2H]^{2+}$ of the **a)** peptide **1**, **b)** metallopeptide **1-Pd**, **c)** peptide **2** and **d)** metallopeptide **2-Pd**.

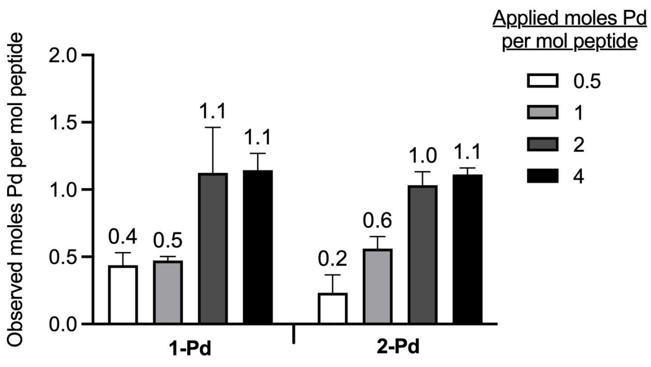

Figure S7. ICP-OES analysis of the resulting metallopeptides **1-Pd** and **2-Pd** (100 μM) in 10% HNO_3 in water after titration molar ratio Pd:peptide 0.5:1; 1:1; 2:1 and 4:1. The molar ratio of Pd to peptide after purification is plotted.

Table S1. HRMS and ICP-OES data of metallopeptides **1-Pd** and **2-Pd**.

	**HRMS $[M+2H]^{2+}$**		**ICP-OES**
	calcd.	found	Pd per metallopeptide (mol%)
**1-Pd** $C_{53}H_{83}N_{11}O_{14}Pd$	601.7572	601.7567	52%
**2-Pd** $C_{56}H_{86}N_{12}O_{16}Pd$	644.2654	644.2650	51%

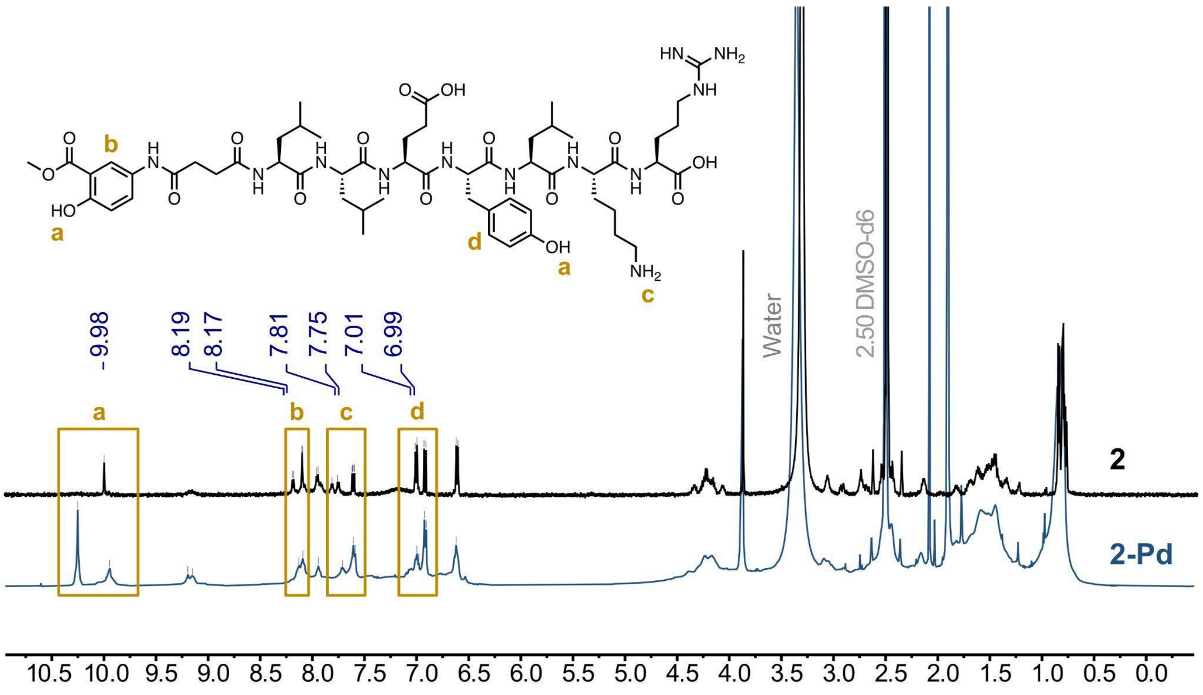

Figure S8. ^{1}H-NMR spectra of peptide **2** (50 mM) without and with $Pd(OAc)_2$ (50 mM) added, assigning proton signal shifts upon complexation in 0.5 mL of DMSO-d_6.

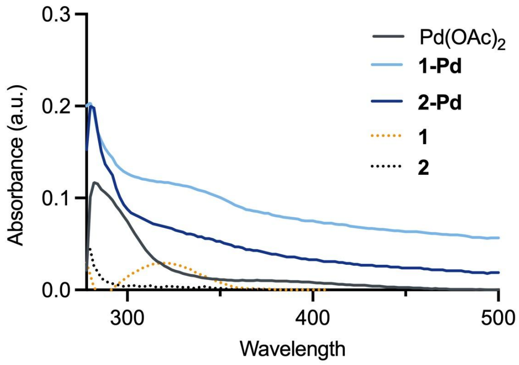

Figure S9. UV-visible spectrum of peptide **1** and **2**, metallopeptide **1-Pd** and **2-Pd**, and $Pd(OAc)_2$ (100 μM) after incubation at 37 °C for 2 h in PBS (1 mL).

3. Determination of the reaction rate constant (K) and half-life ($t_{1/2}$)

Concentrations of product Resorufin (μM) were calculated based on the fluorescence signal of positive control resorufin at 10, 20 and 40 μM, and concentrations of substrate **ProRes** were subtracted from the resulting product Resorufin (μM). The reaction rate constant *K* was estimated by a linear regression of the line plots of Ln[**ProRes**] overtime, providing *K* value equal to slope of the lines and $t_{1/2}$ = Ln (2)/*K*.

a) b)

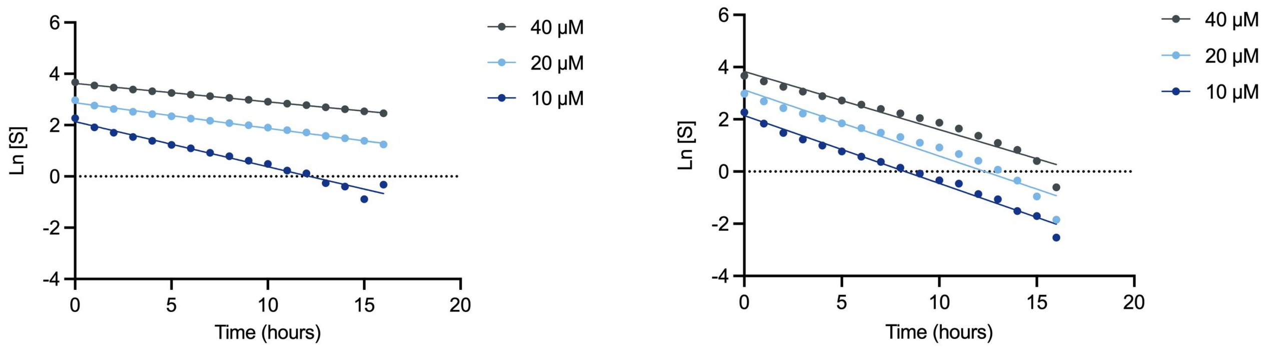

Figure S10. Kinetic study of the reaction by metallopeptides **a) 1-Pd** and **b) 2-Pd** (Pd conc. 5 and 6 μM, respectively) with different concentrations of **ProRes** (10, 20 and 40 μM) in PBS at 37 °C. Natural logarithmic values of the concentration of substrate (**ProRes**) Ln[S] versus time (hours). Curves fit linear regression, r2 > 0.95.

4. Stability studies of the catalyst 2-Pd

a) b)

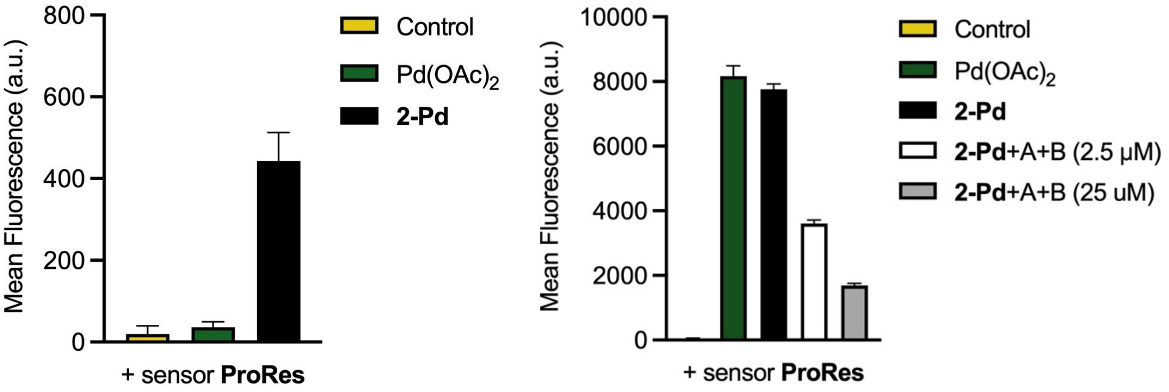

Figure S11. Fluorescence signal for the catalytic conversion of the propargylated resorufin (**ProRes**, 40 μM) into the fluorescent resorufin after incubation at 37 °C for 18 h by **a)** Pd-peptide **2-Pd** (Pd conc. 6 μM) and $Pd(OAc)_2$ (10 μM) in 10% FBS/PBS; or **b)** Pd-peptide **2-Pd** (Pd conc. 6 μM) in the presence of monomers **A** + **B** (2.5 or 25 μM) and $Pd(OAc)_2$ (10 μM) in PBS.

5. Synthesis of drugs paclitaxel (PTX) and linifanib (LNF) by metallopeptide 2-Pd

a)

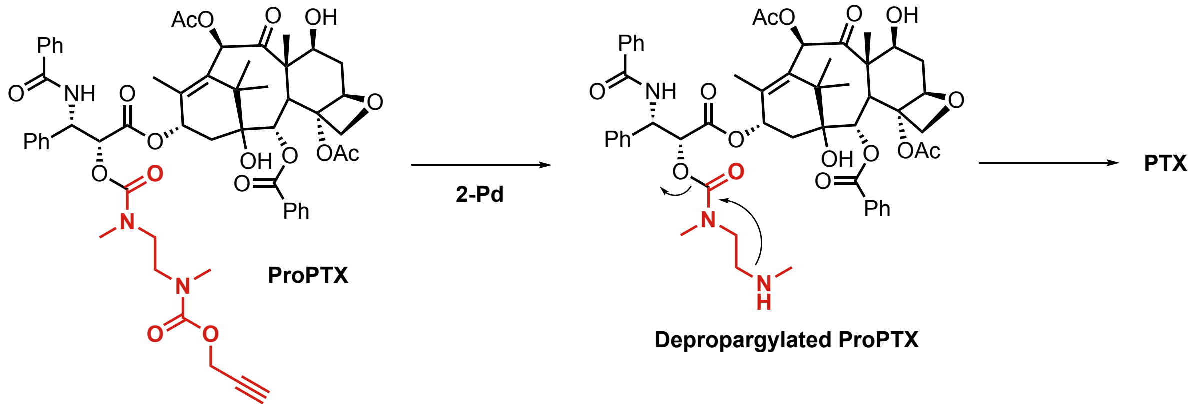

b)

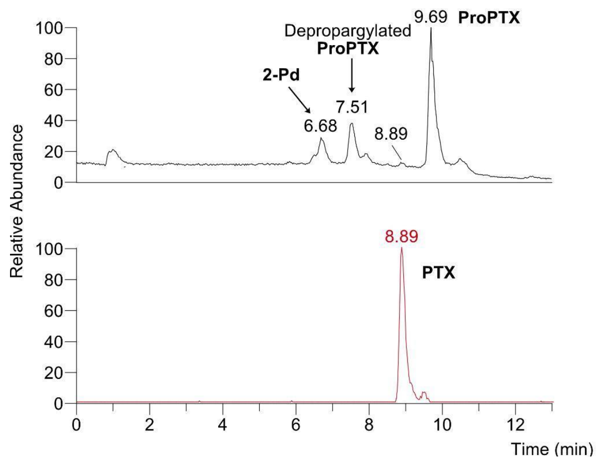

c)

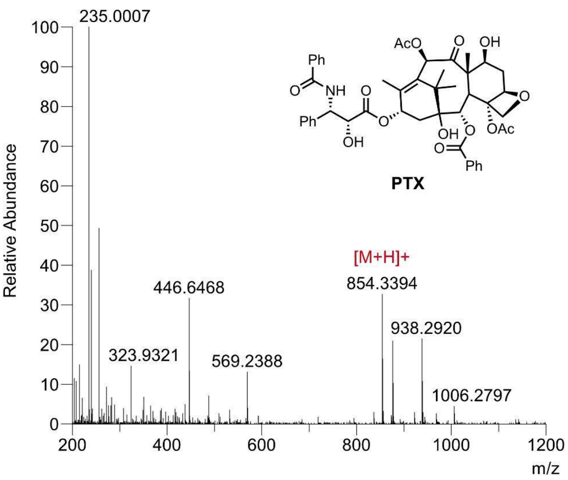

Figure S12. a) Scheme of the Pd-mediated *O*-depropargylation of **ProPTX**.[2] **b-c)** LCMS chromatogram tracking the deprotection reaction of **ProPTX** by metallopeptide **2-Pd** after incubation at 37 °C in PBS for 24 h. **b)** Total ion chromatogram shows metallopeptide **2-Pd**, depropargylated intermediate, **PTX** and **ProPTX**, while **c)** the neutral loss (NL) of **PTX** from the MS identifies the spectra of interest 354.3394 $(M+H)^+$.

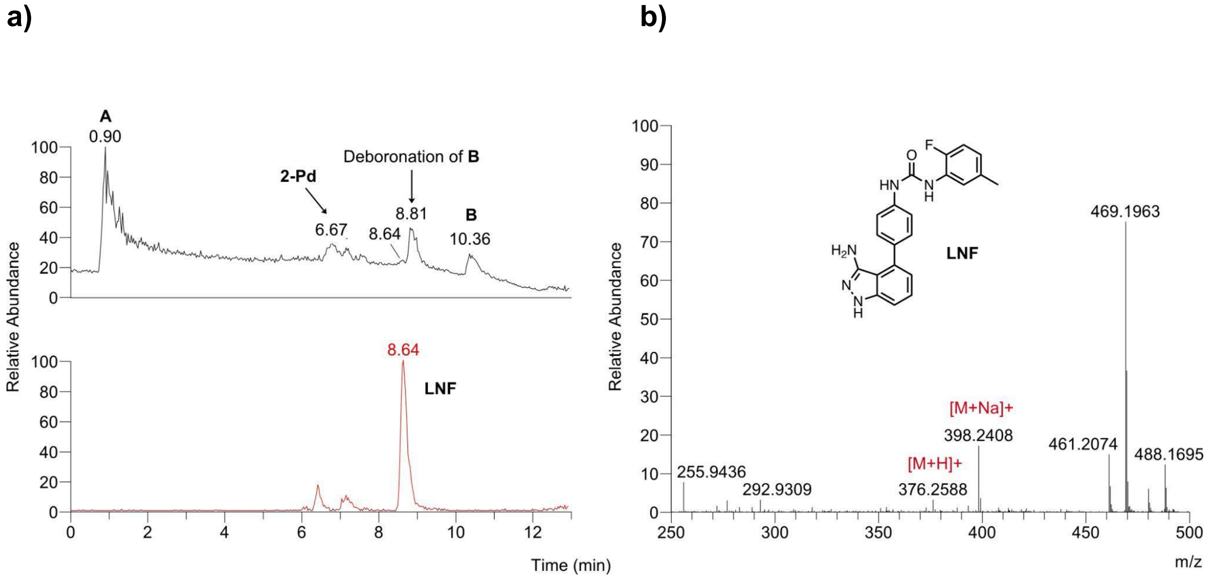

Figure S13. LCMS chromatogram tracking the Suzuki-Miyaura coupling reaction of building blocks **A** and **B** by metallopeptide **2-Pd** after incubation at 37 °C in PBS for 24 h. **a)** Total ion chromatogram shows metallopeptide **2-Pd**, deboronated intermediate, **LNF**, **A** and **B**, while **b)** the neutral loss (NL) of **LNF** from the MS identifies the spectra of interest 398.2408 $(M+Na)^+$.

6. Biological studies

6.1. Synthesis of drugs by metallopeptide 2-Pd

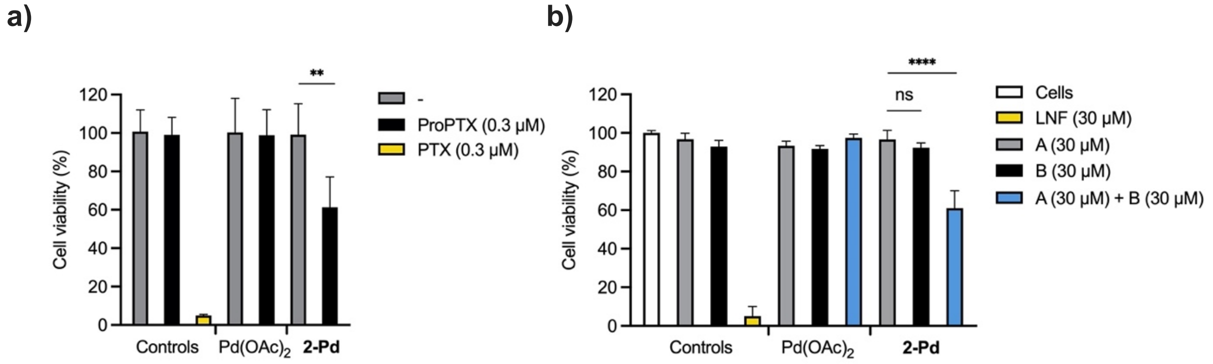

Figure S14. a) Prodrug (**ProPTX**) activation into **PTX** and **b)** Suzuki-Miyaura C-C cross coupling reaction for the synthesis of **LNF** in A549 cells by metallopeptide **2-Pd** (160 μg/mL, Pd conc. 75 μM). Significance was determined by one-way analysis of variance (ANOVA): $^{ns}P > 0.05$, $^{**}P < 0.01$, $^{****}P < 0.0001$.

6.2. Immunofluorescence assay

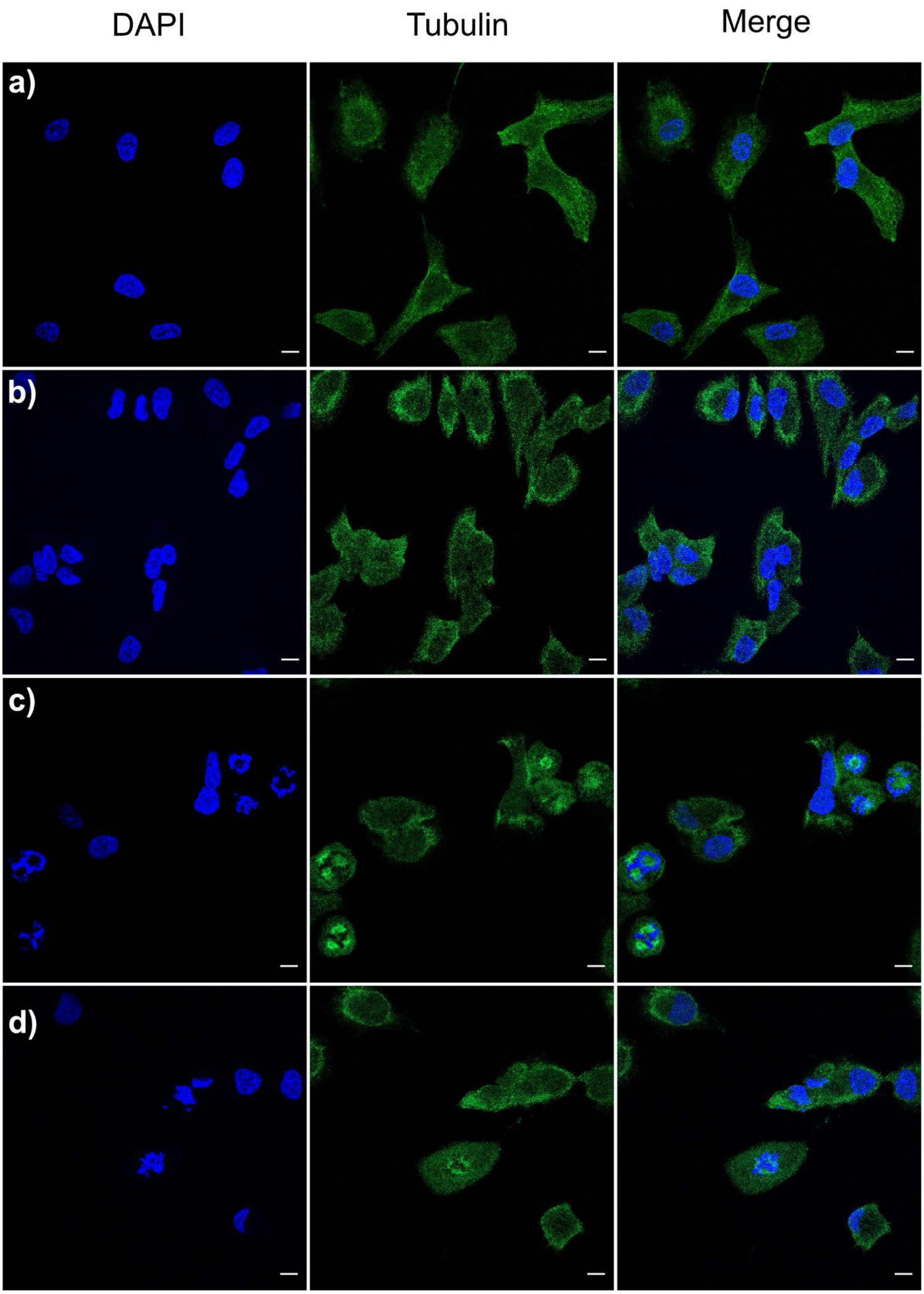

Figure S15. Immunofluorescence study for: **a)** untreated cells; **b)** 0.3 μM **ProPTX** + 30 μM **A** + **B**; **c)** 0.3 μM **PTX** + 30 μM **LNF**; **d)** 160 μg/mL metallopeptide **2-Pd** (Pd conc. 75 μM) + 0.3 μM **ProPTX** + 30 μM **A** + **B** (drug synthesis experiments). 24 h after treatment, cells were fixed and stained with anti-α-tubulin IgG (green) and DAPI (blue). Scale bar= 10 μm.

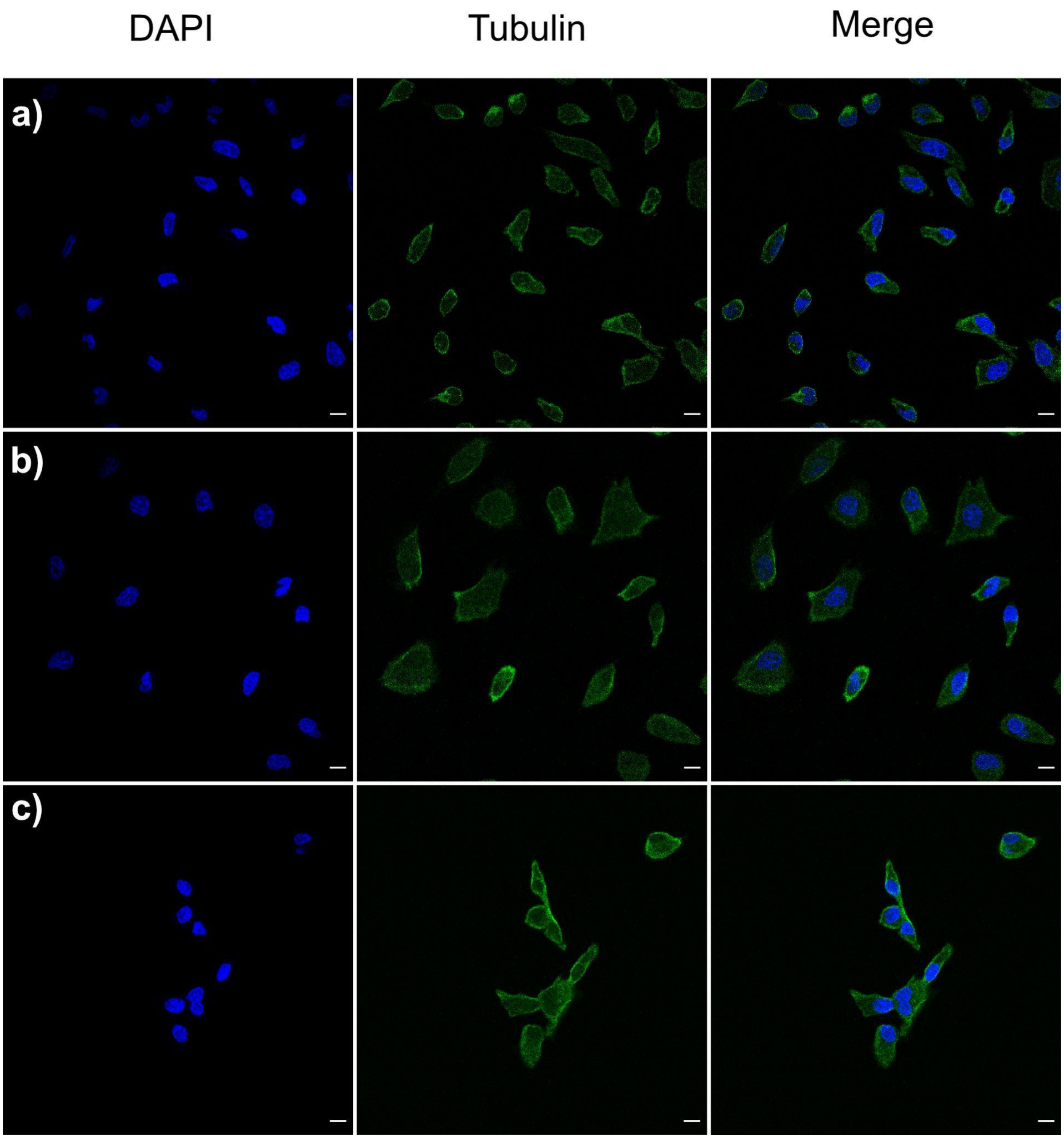

Figure S16. Immunofluorescence study for negative controls: **a)** 160 µg/mL metallopeptide **2-Pd** (Pd conc. 75 µM); **b)** 0.3 µM **ProPTX**; **c)** 30 µM **A** + **B**. 24 h after treatment, cells were fixed and stained with anti-α-tubulin IgG (green) and DAPI (blue). Scale bar= 10 µm.

References

1. Can, M.; Bulut, E.; Özacar, M. Reduction of palladium onto pyrogallol-derived nano-resin and its mechanism. *Chem. Eng. J.* **2015***, 275*, 322–330.

2. Pérez-López, A.M.; Rubio-Ruiz, B.; Valero, T.; Contreras-Montoya, R.; Alvarez de Cienfuegos, L.; Sebastián, V.; Santamaría, J.; Unciti-Broceta, A. Bioorthogonal Uncaging of Cytotoxic Paclitaxel through Pd Nanosheet-Hydrogel Frameworks. *J. Med. Chem.* **2020**, *63*, 9650–9659.
